# Quaternary structure of a G-protein-coupled receptor heterotetramer in complex with G_i_ and G_s_

**DOI:** 10.1186/s12915-016-0247-4

**Published:** 2016-04-05

**Authors:** Gemma Navarro, Arnau Cordomí, Monika Zelman-Femiak, Marc Brugarolas, Estefania Moreno, David Aguinaga, Laura Perez-Benito, Antoni Cortés, Vicent Casadó, Josefa Mallol, Enric I. Canela, Carme Lluís, Leonardo Pardo, Ana J. García-Sáez, Peter J. McCormick, Rafael Franco

**Affiliations:** Centro de Investigación Biomédica en Red sobre Enfermedades Neurodegenerativas (CIBERNED), Madrid, Spain; Institute of Biomedicine of the University of Barcelona (IBUB), Barcelona, Spain; Department of Biochemistry and Molecular Biomedicine, Faculty of Biology, University of Barcelona, Barcelona, 08028 Spain; Laboratori de Medicina Computacional, Unitat de Bioestadística, Facultat de Medicina, Universitat Autònoma de Barcelona, 08193 Bellaterra, Spain; Max Planck Institute for Intelligent Systems, Heisenbergstrasse 3, 70569 Stuttgart, Germany; German Cancer Research Center, Bioquant, Im Neuenheimer Feld 267, 69120 Heidelberg, Germany; Interfaculty Institute of Biochemistry, Hoppe-Seyler-Strasse 4, 72076 Tübingen, Germany; School of Pharmacy, University of East Anglia, Norwich, NR4 7TJ UK

**Keywords:** GPCR, Heterotetramer, Heterotrimeric G protein, Single-particle tracking, BRET, Molecular modeling

## Abstract

**Background:**

G-protein-coupled receptors (GPCRs), in the form of monomers or homodimers that bind heterotrimeric G proteins, are fundamental in the transfer of extracellular stimuli to intracellular signaling pathways. Different GPCRs may also interact to form heteromers that are novel signaling units. Despite the exponential growth in the number of solved GPCR crystal structures, the structural properties of heteromers remain unknown.

**Results:**

We used single-particle tracking experiments in cells expressing functional adenosine A_1_-A_2A_ receptors fused to fluorescent proteins to show the loss of Brownian movement of the A_1_ receptor in the presence of the A_2A_ receptor, and a preponderance of cell surface 2:2 receptor heteromers (dimer of dimers). Using computer modeling, aided by bioluminescence resonance energy transfer assays to monitor receptor homomerization and heteromerization and G-protein coupling, we predict the interacting interfaces and propose a quaternary structure of the GPCR tetramer in complex with two G proteins.

**Conclusions:**

The combination of results points to a molecular architecture formed by a rhombus-shaped heterotetramer, which is bound to two different interacting heterotrimeric G proteins (G_i_ and G_s_). These novel results constitute an important advance in understanding the molecular intricacies involved in GPCR function.

**Electronic supplementary material:**

The online version of this article (doi:10.1186/s12915-016-0247-4) contains supplementary material, which is available to authorized users.

## Background

G-protein-coupled receptor (GPCR) oligomerization is heavily supported by recent biochemical and structural data [1–6]. Optical-based techniques are instrumental in studying the dynamics and organization of receptor complexes in living cells [7]. For instance, total internal reflection fluorescence microscopy shows that 30 % of muscarinic M1 receptors exist as dimers (with no evidence of higher oligomers) that undergo interconversion with monomers on a timescale of seconds [8]. Similarly, the β_1_-adrenergic receptors (β_1_-AR) are expressed as a mixture of monomers and dimers whereas β_2_-adrenergic receptors (β_2_-AR) have a tendency to form dimers and higher-order oligomers [9]. Moreover, the monomer-dimer equilibrium of the chemoattractant *N-formyl* peptide receptor at a physiological level of expression lies within a timescale of milliseconds [10]. Together, these studies in heterologous systems show that a given GPCR is present in a dynamic equilibrium between monomers, dimers, and higher-order oligomers.

Studies in a broad spectrum of GPCRs [11–14] show that these receptors may form heteromers. GPCR heteromers are defined as novel signaling units with functional properties different from homomers and they represent a completely new field of study [15]. Innovative crystallographic techniques have permitted researchers to obtain crystal structures of GPCR families A, B, C, and F, bound to either agonists, antagonists, inverse agonists or allosteric modulators; in the form of monomers or homo-oligomers; and in complex with a G protein or with a ß-arrestin [16]. However, crystal structures of GPCR heteromers have not yet been obtained. Here, we propose a quaternary structure of a heteromer, taking into account the molecular stoichiometry and the interacting G proteins. Adenosine A_1_-A_2A_ receptor (A_1_R-A_2A_R) complexes constitute a paradigm in the GPCR heteromer field because A_1_R is coupled to G_i_ and A_2A_R to G_s_; that is, they transduce opposite signals in cyclic adenosine monophosphate (cAMP)-dependent intracellular cascades. First described as a concentration-sensing device in striatal glutamatergic neurons [17], the A_1_R-A_2A_R heteromer is thought to function as a G_s_/G_i_-mediated switching mechanism by which low and high concentrations of adenosine inhibit and stimulate, respectively, glutamate release [17, 18]. The structural basis of this switch is key to understanding heteromer function and the biological advantage behind the GPCR heteromerization phenomenon. Here, we have devised the molecular architecture of the adenosine A_1_R-A_2A_R heteromer in complex with G proteins using a combination of microscope-based single-particle tracking, molecular modeling, and energy transfer assays in combination with molecular complementation. The results point to A_1_ and A_2A_ receptors organizing into a rhombus-shaped heterotetramer that couples to G_i_ and G_s_. The overall structure is very compact and provides interacting interfaces for GPCRs and for G proteins.

## Results and discussion

### Reciprocal restriction of adenosine receptor motion in the plasma membrane

**Fig. 1:**
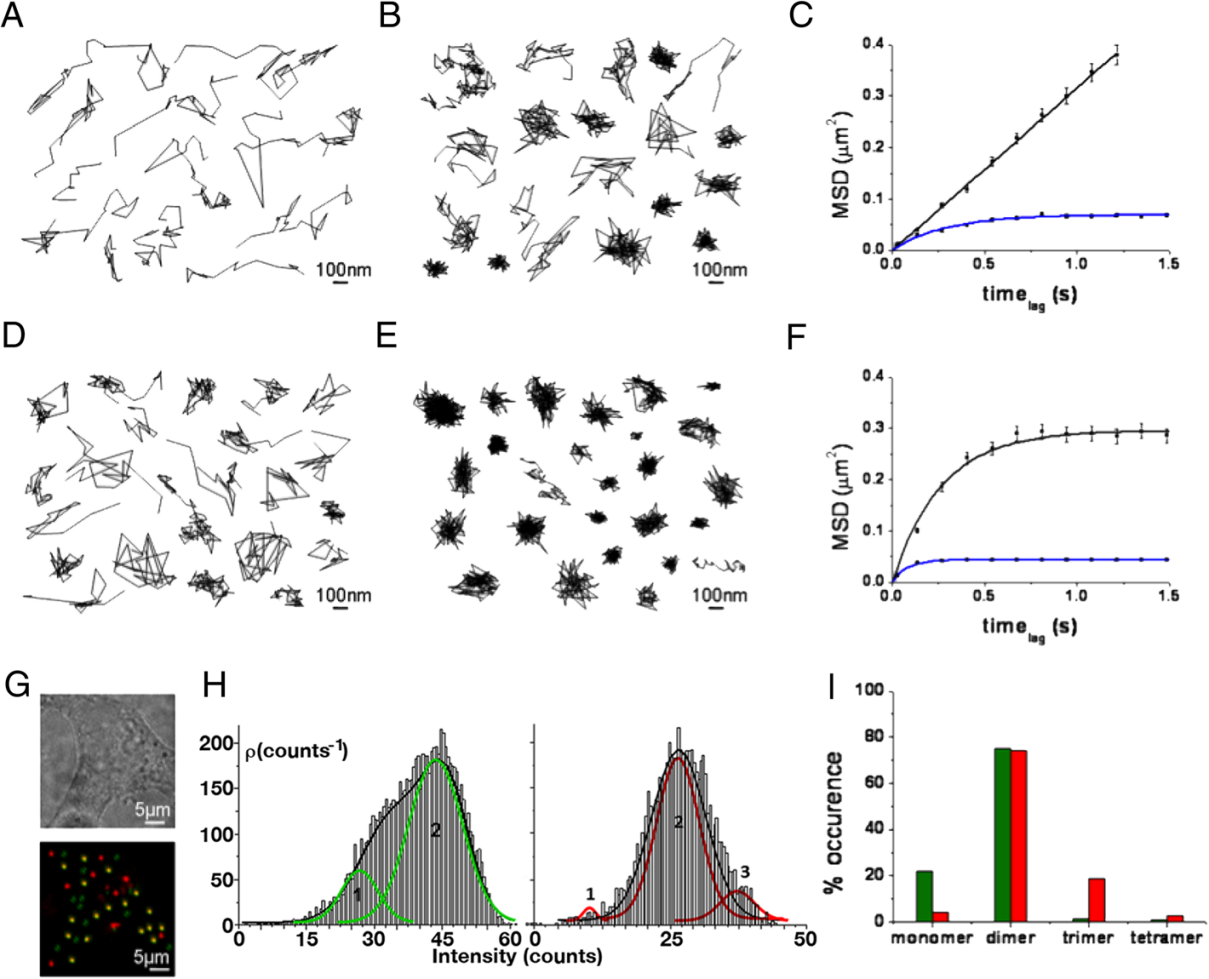
Cell surface mobility of A_1_R-GFP and A_2A_R-mCherry. Individual trajectories of particles containing GFP fused to the C-terminus of A_1_R (A_1_-GFP) (**a** and **b**) or mCherry fused to the C-terminus of A_2A_R (A_2A_-mCherry) (**d** and **e**) on HEK-293T cells expressing A_1_-GFP (a), A_2A_-mCherry (d) or both (b and e). The trajectory and the fluorescence intensity of the individual particles were recorded over time using total internal reflection microscopy (TIRFM) and an electron multiplying charged-coupled device (EMCCD) camera recording. Receptor motion was determined by plotting (versus time lag) the mean square displacement (*MSD*) of A_1_-GFP (**c**) in the absence (*black line*) or presence of A_2A_-mCherry (*blue line*), or A_2A_-mCherry (**f**) in the presence (*black line*) or presence of A_1_-GFP (*blue line*). Data sets were fitted to mathematical models of free and confined diffusion for A_1_R and A_2A_R respectively. **g** Co-localization of A_1_-GFP and A_2A_-mCherry is observed (*yellow dots*). Scale bar: 100 nm. **h** Distribution of the fluorescence signal of A_1_-GFP (*left*) and A_2A_-mCherry (*right*) within co-localized receptors (*yellow dots* in g). Curves approximately delineate the number of monomers, dimers, or trimers within the co-localized complex. **i** Stoichiometry analysis performed for co-localized A_1_-GFP and A_2A_-mCherry receptor particles co-expressed in HEK-293T cells (*yellow dots* in g). *Green* corresponds to A_1_-GFP and *red* to A_2A_-mCherry

To examine the dynamics of A_1_R-A_2A_R heteromers in the plasma membrane of a living cell, the motion of the receptors tagged with fluorescent proteins (A_1_R-green fluorescent protein [GFP] or A_2A_R-mCherry) was measured by real-time single-particle tracking (SPT) (Fig. 1). Examples of fluorescent images and individual particle trajectories are shown in Additional file 1: Figure S1. Analysis of data corresponding to 500 A_1_R-GFP particles showed a linear relationship between the mean square displacement (MSD) versus time lag in the trajectories of up to 1600 single fluorescent particles (Fig. 1a, c). This is typical for Brownian diffusion, indicating a lack of restrictions in A_1_R-GFP motion. Co-expression of A_2A_R-mCherry (Fig. 1b) led to a reduction in the lateral mobility of A_1_R-GFP, which became confined to plasma membrane regions of 0.461 ± 0.004 μm in diameter. Its diffusion coefficient decreased from 0.381 ± 0.002 μm^2^/s to 0.291 ± 0.003 μm^2^/s (*p* = 0.002, one-tailed t-test). Similarly, A_1_R-GFP also decreased the A_2A_R-mCherry diffusion coefficient from 0.317 ± 0.002 μm^2^/s to 0.143 ± 0.005 μm^2^/s (*p* < 0.0001) (Fig. 1d–f). A_2A_R moved within a confinement zone of 0.941 ± 0.007 μm in diameter that was reduced to 0.360 ± 0.001 μm (*p* < 0.0001) when both receptors were co-expressed. We conclude from these mobility comparisons that reciprocally restricted motion of the individual receptor particles must be due to A_1_R-A_2A_R receptor-receptor interactions.

### Stoichiometry of A_1_ and A_2A_ receptor heterocomplexes

The stoichiometry of the fluorescent receptors on the cell surface can be calculated from the brightness distribution of the individual particles [19] (see “Methods”). In cells expressing A_1_R-GFP, we found the majority of clusters to consist of either two (~47 %) or four (~34 %) receptors, and clusters with one or three receptors were scarce (~10 % and ~9 %, respectively) (Additional file 2: Figure S2A and black bars in Additional file 2: Figure S2C). In the case of A_2A_R-mCherry, the stoichiometry analysis showed that the clusters mostly expressed trimers (45 %), with dimers (29 %) and tetramers (12 %) the second and third most common populations (Additional file 2: Figure S2D and black bars in Additional file 2: Figure S2F). Remarkably, this stoichiometry for either A_1_ or A_2A_ receptors was altered when the partner receptor was also expressed. In cells co-expressing A_1_R-GFP and A_2A_R-mCherry, the dimer population increased (57 % for A_1_R-GFP and 49 % for A_2A_R-mCherry, blue bars in Additional file 2: Figures S2C, F) and became the predominant species (Additional file 2: Figures S2B, C, E, F).

In order to focus the analysis on heteromer complexes, we identified clusters containing both receptors (individual yellow dots in Fig. 1g, displaying both GFP and mCherry fluorescence). In ~1000 analyzed co-localized clusters that consisted of a mixture of A_1_-GFP and A_2A_-Cherry (yellow dots in Fig. 1g), we found a similar high amount of dimers of A_1_R (75 %, left panel in Fig. 1h and green bar in Fig. 1i) and A_2A_R (74 %, right panel in Fig. 1h and red bar in Fig. 1i). Trimers and tetramers of A_1_R, and monomers and tetramers of A_2A_R, were in the minority or negligible (see Fig. 1h, i). In summary, given that the percentage of dimers of either A_1_R-GFP or A_2A_R-mCherry in the yellow dots (which show co-localization of the two receptors) was similar and high (~75 %), the heterotetramer containing two A_1_Rs and two A_2A_Rs must have been the most predominant species. To our knowledge, this is the first stoichiometry data for a GPCR heteromer in living cells.

### Arrangement of G proteins interacting with A_1_ and A_2A_ receptors

**Fig. 2:**
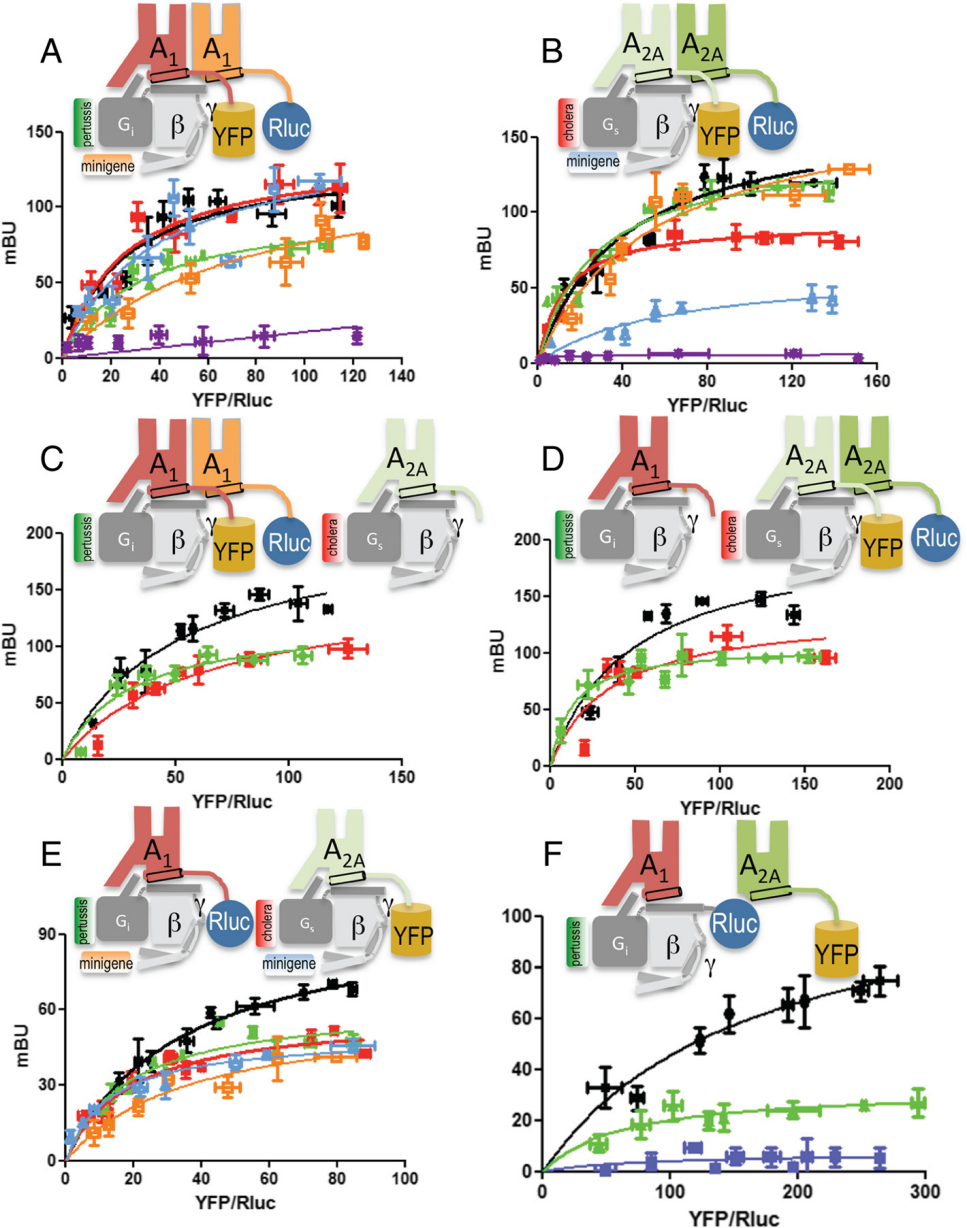
Influence of G proteins on A_1_R and A_2A_R homodimerization and heterodimerization. B Bioluminescence resonance energy transfer (BRET) saturation curves were performed in HEK-293T cells 48 h post-transfection with (**a**, **c**) 0.3 μg of cDNA corresponding to A_1_R-Rluc and increasing amounts of A_1_R-YFP (0.1–1.5 μg cDNA) or GHS1a-YFP (0.25–2 μg cDNA) as negative control (a, *purple line*), without (a) or with (c) 0.15 μg of cDNA corresponding to A_2A_R; (**b**, **d**) 0.2 μg of cDNA corresponding to A_2A_R-Rluc and increasing amounts of A_2A_R-YFP (0.1–1.0 μg cDNA) or GHS1a-YFP (0.25–2 μg cDNA) as negative control (b, *purple line*), without (b) or with (d) 0.5 μg of cDNA corresponding to A_1_R; (**e**) 0.3 μg of cDNA corresponding to A_1_R-Rluc and increasing amounts of A_2AR_-YFP (0.1–1.0 μg cDNA); and (**f**) 0.5 μg of cDNA corresponding to A_1_R (except control *blue curves* that were obtained in cells not expressing A_1_R), 2 μg of cDNA corresponding to G_i_-Rluc, and increasing amounts of A_2A_R-YFP (0.1–0.5 μg cDNA). In panels a, b, and e, cells were also transfected with 0.5 μg of cDNA corresponding to the G_i_-related (*orange curves*) or G_s_-related (*blue curves*) minigenes. Cells were treated for 16 h with medium (*black curves*), with 10 ng/ml of pertussis toxin (*green curves*), or with 100 ng/ml of cholera toxin (*red curves*) prior to BRET determination. To confirm similar donor expressions (approximately 100,000 bioluminescence units) while monitoring the increase in acceptor expression (1000–40,000 fluorescence units), the fluorescence and luminescence of each sample were measured before energy transfer data acquisition. MiliBRET unit (*mBU*) values are the mean ± standard error of the mean of four to six different experiments grouped as a function of the amount of BRET acceptor. In each panel (*top*) a cartoon depicts the proteins to which Rluc and YFP were fused and the presence or not of partner receptors and/or G_s_ or G_i_ proteins [schemes in c to f are not intended to illustrate on stoichiometry because the predominant form in cells expressing the two receptors was the heterotetramer containing two A_1_ and two A_2A_ receptors (see “Results”)]

Monomeric GPCRs are capable of activating G proteins [20]. However, recent findings suggest that one GPCR homodimer bound to a single G protein may be a common functional unit [21]. Thus, an emerging question is how G proteins couple to GPCR heteromers. Because A_1_R selectively couples to G_i_ and A_2A_R to G_s_ [22], the working hypothesis was that both G_i_ and G_s_ proteins may couple to the A_1_R-A_2A_R heterotetramer. To test this hypothesis, we used bioluminescence resonance energy transfer (BRET) assays [23]. In agreement with the SPT experiments (see above), homodimers and heterodimers were detected by BRET assays in cells expressing A_1_R fused with *Renilla* luciferase (A_1_R-Rluc) or yellow fluorescent protein (A_1_R-YFP) (Fig. 2a), A_2A_R-Rluc and A_2A_R-YFP (Fig. 2b), or A_1_R-Rluc and A_2A_R-YFP (Fig. 2e). Neither A_1_R-Rluc nor A_2A_R-YFP interacted with the ghrelin receptor 1a fused to YFP (GHS1a-YFP), used as a control as a protein unable to directly interact with these adenosine receptors (Fig. 2a, b). In order to test the presence of the two G proteins in the heterotetramer, we transfected cells with minigenes that code for peptides blocking either G_i_ or G_s_ binding to GPCRs [24]. In addition, cells were treated with pertussis or cholera toxins that catalyze ADP-ribosylation of G_i_ or G_s_. Clearly, treating cells with pertussis toxin, or expressing the minigene-coded peptide that blocks α_i_ coupling, reduced the value of BRET_max_ for A_1_R-A_1_R homodimers (Fig. 2a) and for A_1_R-A_2A_R heterodimers (Fig. 2e) but not for A_2A_R-A_2A_R homodimers (Fig. 2b). This indicates that G_i_ is coupled to A_1_R in both the homodimer and the heterodimer. Similarly, blocking G_s_-receptor interaction using cholera toxin or a minigene-coded peptide that blocks α_S_ coupling reduced BRET_max_ for A_2A_R-A_2A_R homodimers (Fig. 2b) and for A_1_R-A_2A_R heterodimers (Fig. 2e) but not for A_1_R-A_1_R homodimers (Fig. 2a). Interestingly, BRET curves showed sensitivity to both cholera and pertussis toxins in cells expressing either A_1_R-Rluc-A_1_R-YFP and A_2A_R (Fig. 2c) or A_2A_R-Rluc-A_2A_R-YFP and A_1_R (Fig. 2d). Functionality of constructs and controls in cells expressing minigenes, and in cells expressing the ghrelin GHS1a receptor instead of one of the adenosine receptors, are shown in Additional file 3: Figure S3. To further confirm that G_i_ binds A_2A_R in the receptor heteromer, the energy transfer between Rluc fused to the N-terminal domain of the α-subunit of G_i_ (G_i_-Rluc) and A_2A_R-YFP was analyzed in cells co-expressing or not co-expressing A_1_R (Fig. 2f). A hyperbolic BRET curve was observed in the presence of A_1_R, but not in its absence, indicating that G_i_ and G_s_ are bound to their respective receptor homodimers within the A_1_R-A_2A_R heteromer.

**Fig. 3:**
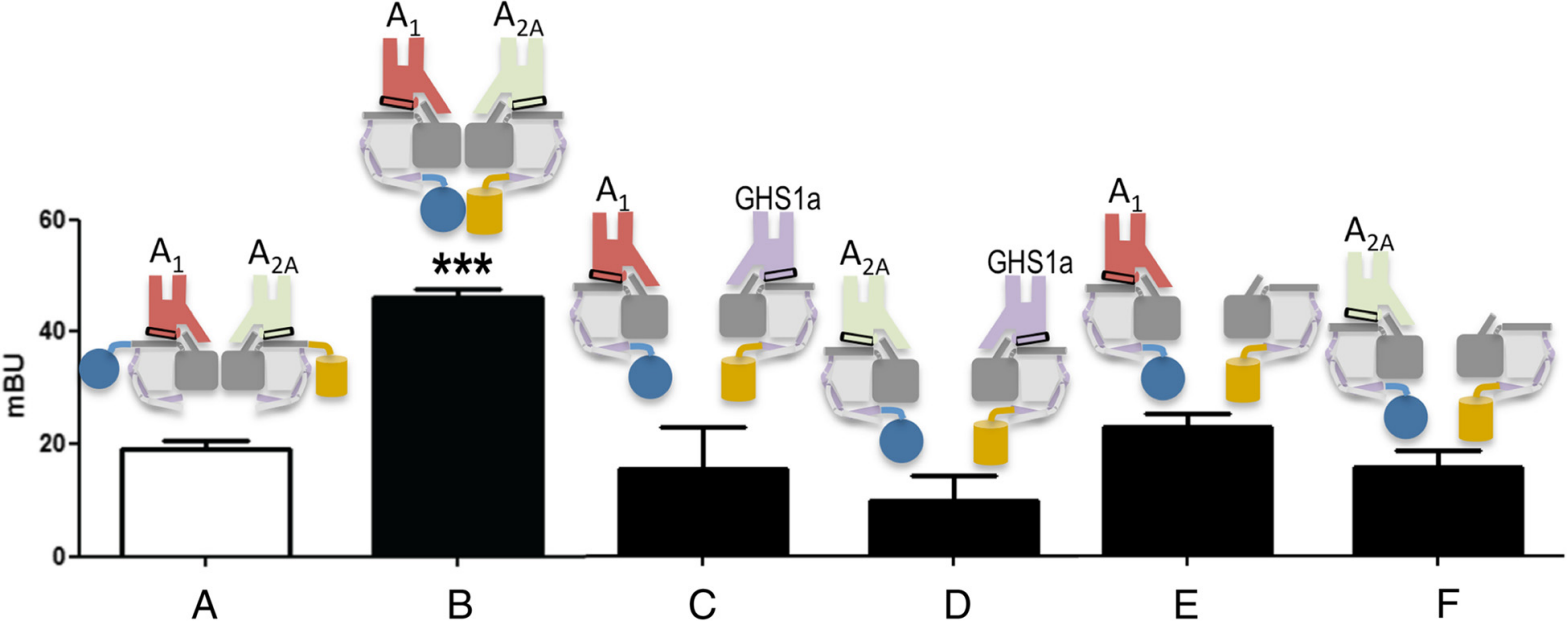
G_s_ and G_i_ coupling to adenosine A_1_R-A_2A_R heterocomplexes. Bioluminescence resonance energy transfer (BRET) experiments were performed in HEK-293T cells 48 h post-transfection with (**a**, **b**) 0.2 μg of cDNA corresponding to A_1_R and 0.15 μg of cDNA corresponding to A_2A_R; (**c**, **d**) 0.2 μg of cDNA corresponding to A_1_R or 0.15 μg of cDNA corresponding to A_2A_R and 0.4 μg of cDNA corresponding to growth hormone secretagogue receptor GHS1a; (**e**) 0.2 μg of cDNA corresponding to A_1_R; or (**f**) 0.15 μg of cDNA corresponding to A_2A_R. Cells were also transfected with 2 μg of cDNA corresponding to the α-subunit of G_i_ fused to Rluc and increasing amounts of cDNA corresponding to the α-subunit of G_s_ fused to YFP (a) or 0.3 μg of cDNA corresponding to the γ-subunit fused to Rluc and increasing amounts of cDNA corresponding to the γ-subunit fused to YFP (b–f). Maximum miliBRET unit (*mBU*) values are the mean ± standard error of the mean of four different experiments. A scheme showing the protein to which Rluc and YFP were fused is provided (*top*). ***p < 0.001 by one-way ANOVA with post - hoc Dunnett’s test

Further, two complementary BRET experiments were performed to determine the orientation of G_i_ and G_s_ within the A_1_R-A_2A_R heterocomplex. First, Rluc and YFP were respectively fused to the N-terminal domains of the α-subunit of G_i_ (α_i_-Rluc) and G_s_ (α_s_-YFP) (Fig. 3, bar a); second, they were fused to the N-terminal domain of the γ-subunit (γ-Rluc and γ-YFP) (Fig. 3, bar b). We observed significant energy transfer between γ-Rluc and γ-YFP in cells co-expressing A_1_R and A_2A_R (Fig. 3, bar b) but minimal amounts in negative-control cells (Fig. 3, bars c and d). In cells expressing either A_1_R or A_2A_R, the energy transfer between γ-Rluc and γ-YFP was also low (Fig. 3, bars e and f), suggesting that dimers but not tetramers were the most prevalent form of surface receptors in single-transfected cells. These results in co-transfected cells corroborate the 2:2 stoichiometry obtained from analysis of the fluorescence in single particles and are consistent with G_i_ and G_s_ binding to these A_1_R-A_2A_R heterotetramers.

### Molecular model of G_i_ and G_s_ bound to the A_1_R-A_2A_R heterotetramer

**Fig. 4:**
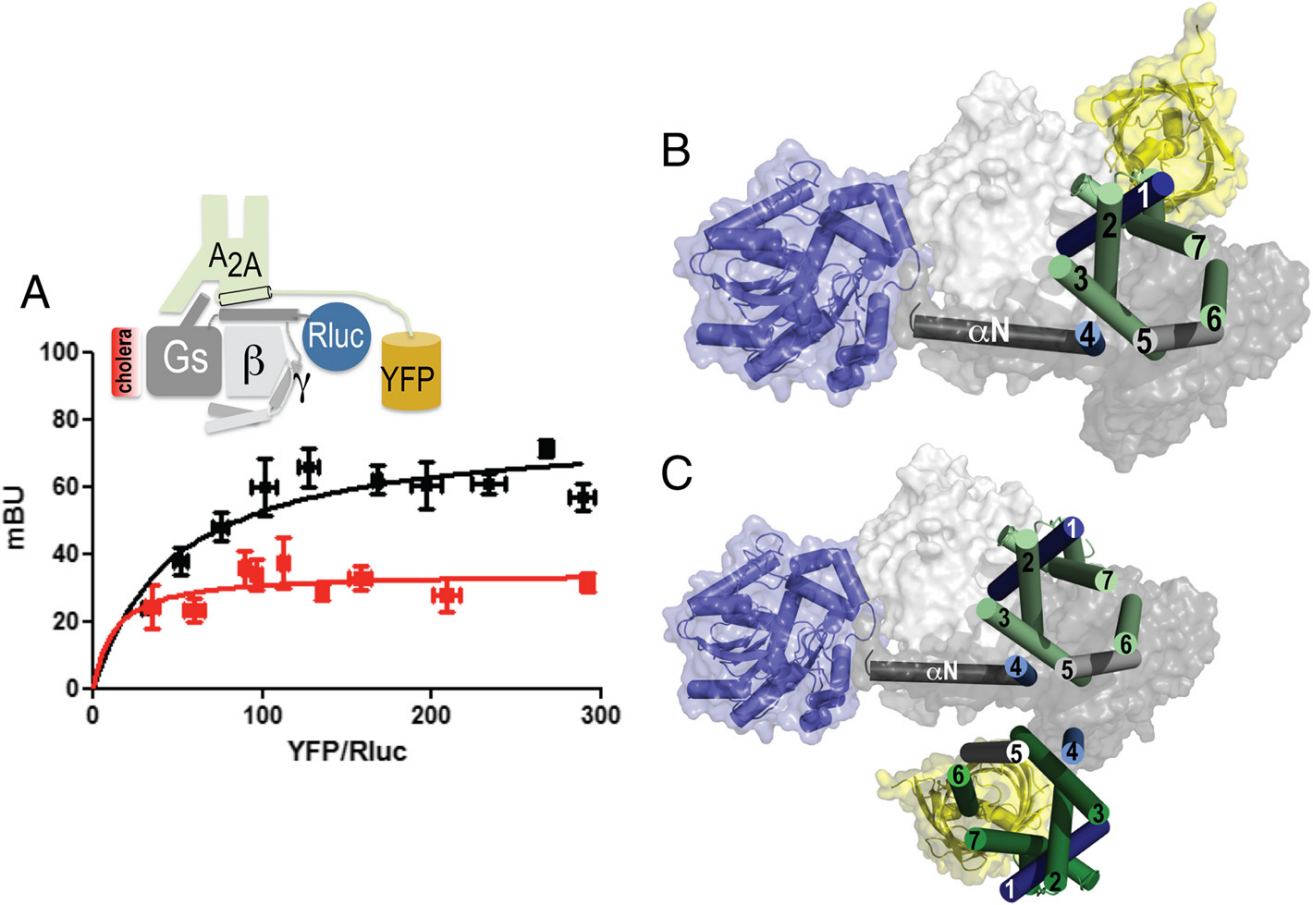
Orientation of a G protein in a receptor homodimer. Bioluminescence resonance energy transfer (BRET) saturation experiments were performed in HEK-293T cells transfected with 2 μg of cDNA corresponding to the α-subunit of G_s_ fused to Rluc and increasing amounts of A_2A_R-YFP (0.1–0.5 μg) cDNA. **a** BRET measurements in cells pretreated for 16 h with medium (*black line*) or with 100 ng/ml of cholera toxin (*red line*). Both fluorescence and luminescence of each sample were measured before every experiment to confirm similar donor expressions (approximately 50,000 bioluminescence units) while monitoring the increase in acceptor expression (1000–10,000 fluorescence units). miliBRET unit (*mBU*) values are the mean ± standard error of the mean of four to five different experiments grouped as a function of the amount of BRET acceptor. A scheme of the placement of donor and acceptor BRET moieties is provided (*top*). **b** Molecular model of the A_2A_R-G_s_ complex. Rluc (*blue*) is attached to the N-terminal αN helix of G_s_ (*gray*), and YFP (*yellow*) is attached to the C-terminal domain of A_2A_R (*light green*) (see Additional file 9: Figure S9 for details). **c** Arrangement of A_2A_R homodimers modeled via the TM4/5 interface as observed in the oligomeric structure of β_1_-AR [4]. The A_2A_R protomer bound to α_s_ is shown in *light green*, whereas the second A_2A_R-YFP protomer is shown in *dark green*. The molecular model in panel c (BRET between Rluc in G_s_ α subunit and YFP in a second A_2A_R protomer; center-to-center distance between Rluc and YFP of 6.5 nm), in contrast to the model shown in panel B (BRET between Rluc in G_s_ α subunit and YFP in the G-protein bound A_2A_R protomer; center-to-center distance between Rluc and YFP of 8.3 nm), would favor the observed high-energy transfer (see panel a) between α_s_-Rluc and A_2A_R-YFP

To identify the orientation of the G protein in the receptor homodimer, we combined energy transfer assays between α_s_-Rluc (Rluc at the N-terminus of the G protein α-subunit) and A_2A_R-YFP (Fig. 4a) with information on transmembrane (TM) interfaces based on crystal structures of GPCRs [3, 4], which have been recently summarized [25]. The observed high-energy transfer using α_s_-Rluc and A_2A_R-YFP indicated close proximity between the N-tail of the α-subunit of G_s_ and the C-tail of A_2A_R. Interestingly, Rluc and YFP in the “monomeric” A_2A_R-G_s_ complex (see “Methods”) point toward distant positions in space (Fig. 4b). Therefore, the observed BRET should occur between Rluc in the G protein α-subunit and a second A_2A_R-YFP protomer. Among all described TM interfaces for receptor homodimerization (see Additional file 4: Figure S4), we propose the TM4/5 interface, which is observed in the oligomeric structure of β_1_-AR [4] and in structures derived from coarse-grained molecular dynamics (MD) simulations [26]. In fact, this is the only interface that favors BRET between α_s_-Rluc and a second A_2A_R-YFP protomer in a homodimer (Fig. 4c). The homologous A_1_R homodimer was built using the same TM4/5 interface as for A_2A_R (see Additional file 4: Figure S4 and its legend).

**Fig. 5.**
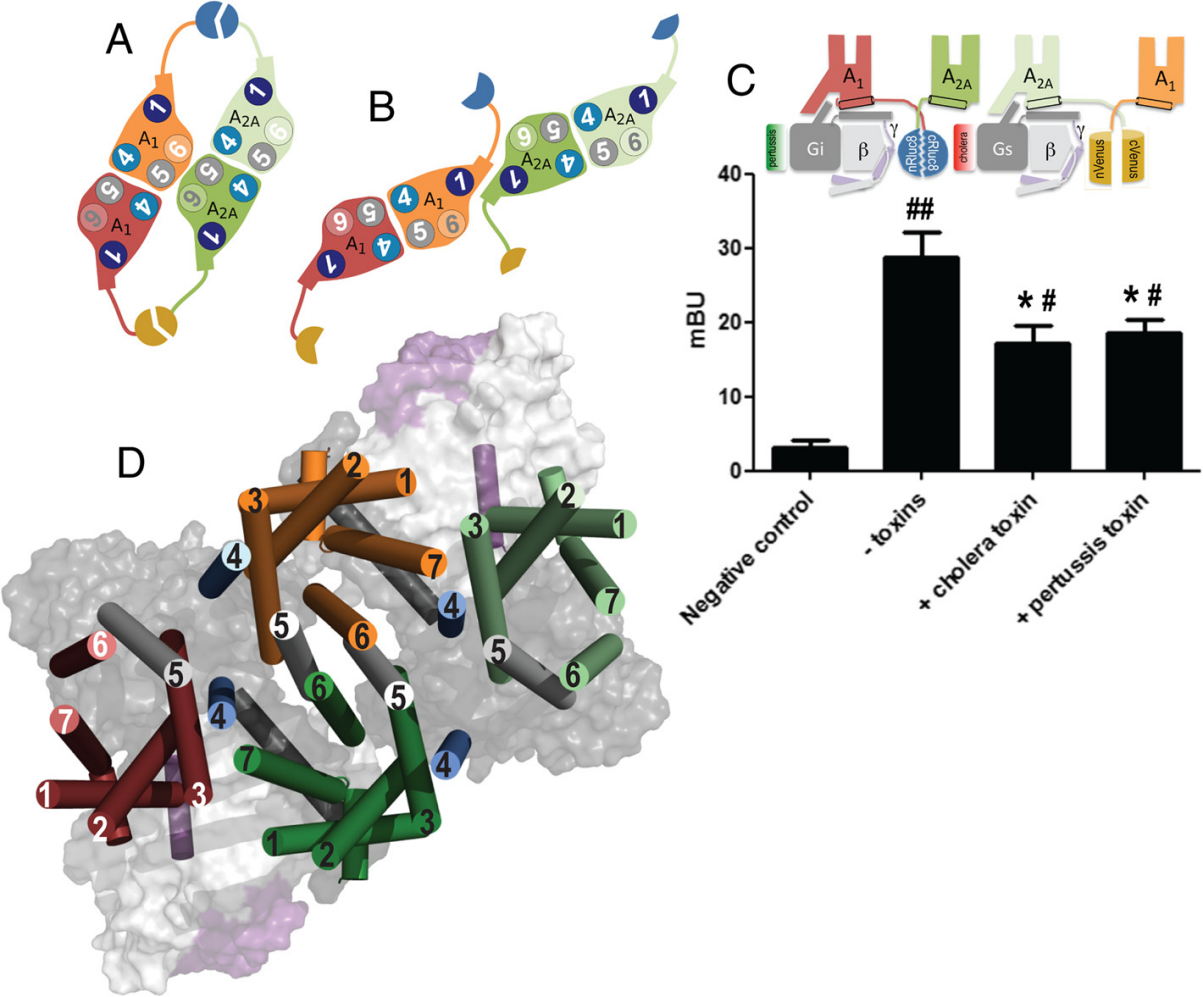
Bioluminescence resonance energy transfer (BRET)-aided construction of a model consisting of G_i_ and G_s_ bound to an A_1_R-A_2A_R heterotetramer. **a**, **b** A_1_R-A_2A_R tetramer built using TM5/6 (a) or TM1 (b) inter-receptor interfaces modeled as in the structure of the μ opioid receptor [3]. TM helices 1, 4, and 5, involved in receptor dimerization, are highlighted in *dark blue*, *light blue*, and *gray*, respectively. nRluc8 and cRluc8 are shown in *blue* and nVenus and cVenus in *dark yellow*. **c** BRET and bimolecular fluorescence complementation experiments were performed in HEK-293T cells transfected with 1.5 μg of cDNA corresponding to A_1_R-cRluc8 and A_2A_R-nRluc8, and 1.5 μg of cDNA corresponding to A_1_R-nVenus and A_2A_R-cVenus. As the negative control, cells were transfected with 1 μg of cDNA corresponding to nRluc8 and 1.5 μg of cDNA corresponding to A_2A_R-nRluc8, A_1_R-nVenus, and A_2A_R-cVenus. Cells were treated for 16 h with medium (*– toxins*), 10 ng/ml of pertussis toxin (*+ pertussis*), or 100 ng/ml of cholera toxin (*+ cholera*) prior to BRET determination. The relative amount of BRET is given as in Fig. 4 and values are the mean ± standard error of the mean of three different experiments. Student’s t-test showed statistically significant differences with respect to the control (^#^ *p* < 0.05, ^##^ *p* < 0.01) and a significant effect in the presence of either toxin over BRET in the absence of toxins (*p < 0.05). A schematic representation at the *top* shows the protein to which the hemi luminescent or fluorescent proteins were fused. **d** Molecular model of the A_1_R-A_2A_R tetramer in complex with G_i_ and G_s_. A_1_R bound to G_i_ is shown in *red*, G_i_-unbound A_1_R is shown in *orange*, A_2A_R bound to G_s_ is shown in *dark green*, G_s_-unbound A_2A_R is shown in *light green*, and the α, β-, and γ-subunits of G_i_ and G_s_ are shown in *dark gray*, *light gray*, and *purple*, respectively. Transmembrane helices 4 and 5 are highlighted in *light blue* and *gray*, respectively

The remaining possible TMs able to form heteromeric interfaces are TM1 and TM5/6 (Fig. 5). Both are possible inter-GPCR interfaces as observed in the structure of the μ-opioid receptor (μ-OR) [3]. To discern between these two possibilities, a bimolecular fluorescence complementation strategy was undertaken. For this purpose, the N-terminal fragment of Rluc8 was fused to A_1_R (A_1_R-nRluc8) and its C-terminal domain to A_2A_R (A_2A_R-cRluc8), which only upon complementation can act as a BRET donor (Rluc8). The BRET acceptor protein was obtained upon complementation of the N-terminal fragment of YFP Venus protein fused to A_1_R (A_1_R-nVenus) and its C-terminal domain fused to A_2A_R (A_2A_R-cVenus). When all four receptor constructs were transfected, we obtained a positive and saturable BRET signal (BRET_max_ of 35 ± 2 mBU and BRET_50_ of 16 ± 3 mBU) that was not obtained for negative controls (Additional file 5: Figure S5). Figure 5a, b shows that the hemi-donor (A_1_R-nRluc8 and A_2A_R-cRluc8) and the hemi-acceptor (A_1_R-nVenus and A_2A_R-cVenus) moieties, placed at the C-terminus of the receptors, can only complement if A_1_R-A_2A_R heterodimerization occurs via the TM5/6 interface. The TM4/5 interface for homodimerization and the TM5/6 interface for heterodimerization give a rhombus-shaped tetramer organization (Fig. 5a). Remarkably, cell pre-incubation with either pertussis or cholera toxins decreased the BRET_max_ by 35 % (Fig. 5c), further suggesting that both G_s_ and G_i_ proteins bind to the A_1_R-A_2A_R heterotetramer.

We next evaluated, using computational tools, whether the proposed A_1_R-A_2A_R heterotetramer could couple to both G_i_ and G_s_ proteins. Clearly, the external protomers of the proposed A_1_R-A_2A_R heterotetramer can bind to G_i_ and G_s_ proteins (Fig. 5d). This model positions the α-subunits of G_i_ and G_s_ in close contact, facing the interior of the tetrameric complex, while the N-terminal α-helices of α_i_ and α_s_ point outside the complex. The N-terminal α-helices of the γ-subunits are in close proximity, facing the inside (Additional file 6: Figure S6), which explains the significant energy transfer observed between γ-Rluc and γ-YFP (Fig. 3, bar b). The model provides experimental insights into the structural arrangement of heteromers consisting of two GPCRs and coupled to two G proteins, the possibility of which has recently been discussed [25]. We used MD simulations to study the stability of this complex. Additional file 7: Figure S7 shows root-mean-square deviations (rmsd) on protein α-carbons throughout the MD simulation, as well as key intermolecular distances among protomers and G proteins. Clearly, both the A_1_R protomer bound to G_i_ and the A_1_R protomer that does not interact with it maintained a close structural similarity (rmsd ≈ 0.3 nm) relative to the initial structures. Similar results were obtained for the A_2A_R protomers (bound and unbound to G_s_) (Additional file 7: Figure S7A). The fact that rmsd values of the whole system, formed by the A_1_R-A_2A_R heterotetramer bound to G_i_ and G_s_, are of the order of 0.6 nm indicates that the initial structural model is maintained during the MD simulation (Additional file 7: Figure S7A). As a consequence, selected intermolecular distances among protomers and G proteins remain constant during the MD simulation (Additional file 7: Figure S7B). A key aspect in the assembly of the heterotetramer is the TM interfaces for homodimerization (TM4/5) and heterodimerization (TM5/6). Additional file 8: Figure S8B shows rmsd values of the four-helix bundle forming the TM4/5 and TM5/6 interfaces, the initial and final snapshots of these bundles, and the evolution of the A_1_R-A_2A_R heterotetramer during the MD simulation. Clearly, the rather small structural variations of these four-helix bundles, also reflected by rmsd <0.3 nm, suggest a stable complex. Notably, the TM5/6 four-helix bundle seems more stable than the TM4/5 bundles, as shown by its lower rmsd value. Additional file 8: Figure S8B, C depicts contact maps of the TM4/5 and TM5/6 interfaces, as well as the evolution of the network of hydrophobic interactions within these interfaces during the MD simulation.

## Conclusions

For more than a decade, experimental evidence has supported the occurrence of homo-oligomers and hetero-oligomers of GPCRs [21]. However, our basic understanding of what makes heteromers different from homomers remains unknown. Our results, studying adenosine receptors as a model heteromer, point to three important new findings. First, the predominant stoichiometry in cells expressing A_1_R-A_2A_R heteromers is 2:2; that is, a dimer of dimers (tetramer). Second, two different heterotrimeric G proteins can couple to heteromers, the overall complex constituting a functional unit. Third, the molecular orientation within the heteromer complex affords various qualitatively different interfaces; the two more relevant are the inter-protomer heteromeric interface and the inter-G-protein interface. Presumably, the two interfaces provide the key characteristic of heteromers: the ability of one protomer/G-protein complex to influence the signaling of the other. Surely, allosteric effects occurring between heteroreceptors and between G_s_ and G_i_ proteins are due to conformational changes transmitted along the intimately interacting molecules in the complex. In our controlled cell transfection system, which expressed a low density of receptors, minor species formed by monomers and trimers were found in addition to a predominance of tetramers in the plasma membrane, strongly supporting the occurrence of an in vivo dynamic distribution of receptors.

Adenosine was, from an evolutionary point of view, one of the first extracellular regulators given that it is involved in energy and nucleic acid metabolisms. Adenosine A_1_ and A_2A_ receptors are expressed in almost every mammalian organ and tissue. In the heart, where adenosine plays a key role in both inotropic and chronotropic regulation, A_1_R-mediated cardioprotection did not occur in A_2A_R knockout mice, suggesting an interaction between A_1_ and A_2A_ receptors. In neurons, A_1_ and A_2A_ receptors show co-localization, leading to inter-receptor interactions unveiled by pharmacological treatments. For instance, Okada et al. [27] showed that cAMP-dependent protein kinase A plays a role in the regulation of hippocampal serotonin release mediated by both A_1_ and A_2A_ receptors. Similarly, the control of γ-amino butyric acid transport in astrocytes was attributed to the expression of A_1_R-A_2A_R heteromers and to a specific mechanism by which the heteromer signals via G_i_ or G_s_ depending on the concentration of adenosine [28]. The structural basis of the differential signaling by the heteromer/G-protein macromolecular complex likely implies communication at the receptor-receptor level but also between G_s_ and G_i_. Because the binding of two G proteins to a heterodimer is not feasible due to steric clashes [25], our finding that the A_1_R-A_2A_R heterotetramer may bind to both G_s_ and G_i_ provides a structural framework to interpret experimental data.

## Methods

### Total internal reflection microscopy and single-particle data analysis

Single-particle imaging and tracking were performed on a Nikon Total Internal Reflection Fluorescence (TIRF) system, as detailed in Additional file 11: Supplementary Methods. Typically, 500 readouts of a 512 × 512-pixel region, the full array of the CCD chip, were acquired. For single-particle data analysis, parameters were calculated by applying the equations described in Additional file 11: Supplementary Methods.

### Cell culture and transient transfection

HEK-293T cells were grow at 37 °C in Dulbecco’s modified Eagle’s medium (DMEM) (Gibco, Thermo Fischer Scientific, Madrid, Spain) supplemented with 2 mM L-glutamine, 100 U/ml penicillin/streptomycin, and 5 % (v/v) heat-inactivated fetal bovine serum (FBS) (all supplements were from Invitrogen, Paisley, UK). Cells were transiently transfected with cDNA corresponding to receptors, fusion proteins, A_2A_R mutants, or G-protein minigene vectors obtained as detailed in an expanded view by the polyethylenimine (PEI; SigmaAldrich, Cerdanyola del Vallès, Spain) method. Sample protein concentration was determined using a Bradford assay kit (Bio-Rad, Munich, Germany) using bovine serum albumin dilutions as standards. For single-particle imaging, cells were seeded into six-well plates containing glass coverslips (No. 1, round, 24 mm; Assistent, Sondheim, Germany) or into the Lab-Tek Chambered #1.0 Borosilicate Coverglass System (Nunc, Thermo Fisher Scientific, Schwerte, Germany). Cell transient transfections were performed with Lipofectamine™ 2000 (Invitrogen, Life Technologies, Darmstadt, Germany) or FuGENE 6 (Roche Applied Science, Indianapolis, IN, USA) and the application of 0.1–0.2 μg plasmid DNA per well. Before each experiment, cells were washed three times with 200 μL phenol red-free DMEM.

### Plasmids

DNA sequences encoding amino acid residues 1–155 and 155–238 of YFP Venus protein, and amino acids residues 1–229 and 230–311 of RLuc8 protein were subcloned in the pcDNA3.1 vector to obtain the YFP Venus and RLuc8 hemi-truncated proteins. The human cDNAs for adenosine receptors, A_2A_R and A_1_R, cloned into pcDNA3.1, were amplified without their stop codons using sense and antisense primers harboring unique EcoRI and BamHI sites to clone receptors into the pcDNA3.1RLuc vector (p*RLuc*-N1; PerkinElmer, Wellesley, MA, USA), and EcoRI and KpnI to clone A_2A_R, A_1_R, or GHS1a into the pEYFP-N1 vector (enhanced yellow variant of GFP; Clontech, Heidelberg, Germany). G_αs_ cloned into the *SFV1* vector, G_αi_ cloned into the pcDNA3.1 vector, or G_γ_ cloned into the *pEYFP-C1* vector were amplified without their stop codons using sense and antisense primers harboring unique *Hind*III and *Bam*HI sites to clone them into the pcDNA3.1-Rluc vector, or EcoRI and KpnI to clone G_αs_ into the pEYFP-N1 vector. The amplified fragments were subcloned to be in-frame with restriction sites of the pcDNA3.1RLuc or pEYFP-N1 vectors to give plasmids that expressed proteins fused to RLuc or YFP on the N-terminal end (G_αs_-RLuc, G_αi_-RLuc, G_γ_-RLuc, G_αs_-YFP, and G_γ_-YFP) or the C-terminal end (A_1_R-RLuc, A_2A_R-RLuc, A_1_R-YFP, A_2A_R-YFP, and GHS1a-YFP). The human cDNAs for A_1_R or GHS1a were subcloned into pcDNA3.1-nRLuc8 or pcDNA3.1-nVenus to give plasmids that expressed A_1_R or GHS1a fused to either nRLuc8 or nYFP Venus on the C-terminal end of the receptor (A_1_R-nRLuc8 and A_1_R-nVenus or GHS1a-nRLuc8 and GHS1a-nVenus). The cDNAs for human A_2A_ or GHS1a receptors were subcloned into pcDNA3.1-cRLuc8 or pcDNA3.1-cVenus to give plasmids that expressed receptors fused to either cRLuc8 or cYFP Venus on the C-terminal end of the receptor (A_2A_R-cRLuc8 and A_2A_R-cVenus or GHS1a-cRLuc8 and GHS1a-cVenus). Expression of constructs was tested by confocal microscopy and the receptor-fusion protein functionality by measuring ERK1/2 phosphorylation and cAMP production, as described previously [13, 14, 17, 29].

“Minigene” plasmid vectors are constructs designed to express relatively short polypeptide sequences following their transfection into mammalian cells. Here, we used minigene constructs encoding the carboxyl-terminal 11-amino acid residues from G_α_ subunits of G_i1/2_ (G_i_ minigene) or G_s_ (G_s_ minigene) G proteins; the resulting peptides inhibit G-protein coupling to the receptor and consequently inhibit the receptor-mediated cellular responses as previously described [24]. The cDNA encoding the last 11 amino acids of human G_α_ subunit corresponding to G_i1/2_ (I K N N L K D C G L F) or G_s_ (Q R M H L R Q Y E L L), inserted in a pcDNA3.1 plasmid vector, were generously provided by Dr Heidi Hamm.

### Energy transfer assays

For BRET and complementation BRET assays, HEK-293T cells were transiently cotransfected with a constant amount of cDNA encoding for proteins fused to RLuc, nRLuc8, or cRLuc8, and with increasing amounts of the cDNA corresponding to proteins fused to YFP, nYFP Venus, or cYFP Venus (see figure legends). To quantify protein-YFP expression or protein-reconstituted YFP Venus expression, cells (20 μg protein) were distributed in 96-well microplates (black plates with a transparent bottom) and fluorescence was read in a FLUOstar OPTIMA Fluorimeter (BMG Labtechnologies, Offenburg, Germany) equipped with a high-energy xenon flash lamp, using a 10 nm bandwidth excitation filter at 400 nm reading. Protein fluorescence expression was determined as the fluorescence of the sample minus the fluorescence of cells expressing the BRET donor alone. For BRET measurements, the equivalent of 20 μg of cell suspension were distributed in 96-well microplates (Corning 3600, white plates; Sigma) and 5 μM coelenterazine h (Molecular Probes, Eugene, OR, USA) was added. After 1 min for BRET or after 5 min for BRET with bimolecular fluorescence complementation, the readings were collected using a Mithras LB 940 that allows the integration of the signals detected in the short-wavelength filter at 485 nm (440–500 nm) and the long-wavelength filter at 530 nm (510–590 nm). To quantify protein-RLuc or protein-reconstituted RLuc8 expression, luminescence readings were also performed 10 min after adding 5 μM coelenterazine h. The net BRET was defined as [(long-wavelength emission)/(short-wavelength emission)] – Cf, where Cf corresponds to [(long-wavelength emission)/(short-wavelength emission)] for the donor construct expressed alone in the same experiment. BRET is expressed as miliBRET units (mBU; net BRET × 1000).

### Computational model of the A_1_R-A_2A_R tetramer in complex with G_i_ and G_s_

The crystal structure of inactive A_2A_R [PDB:4EIY] [30] was used for the construction of human A_2A_R [UniProt:P29274] and A_1_R [UniProt:P30542] homology models using Modeller 9.12 [31]. These receptors share 51 % of sequence identity and 62 % of sequence similarity, excluding the C-terminal after helix 8. Intracellular loop 3 (ICL3) of A_2A_R (Lys209–Gly218) and A_1_R (Asn212–Ser219) were modeled using Modeller 9.12 [31] using ICL3 of squid rhodopsin [PDB:2Z73] as a template. The C-terminus tails of A_1_R, containing 16 amino acids (Pro311–Asp326), and of A_2A_R, containing 102 amino acids (Gln311–Ser412), were modeled as suggested for the oxoeicosanoid receptor (OXER) [32] (see Additional file 9: Figure S9 for details). The N-terminus of A_1_R and A_2A_R were not included in the model. The “active” conformations of A_1_R bound to G_i_ and A_2A_R bound to G_s_ were modeled using the crystal structure of β_2_-AR in complex with G_s_ [PDB:3SN6] [33]. The globular α-helical domain of the α-subunit was modeled in the “closed” conformation [34], using the crystal structure of [AlF_4_^−^]-activated G_i_ [PDB:1AGR]. The location of YFP [PDB:2RH7] attached to the C-tail of A_2A_R was determined as suggested for the OXER [32] (see Additional file 9: Figure S9 for details). Rluc [PDB:2PSD] and YFP were fused to the to the N-terminus of the α-subunits and γ-subunits of G_i_ and G_s_ by a covalent bond. The structures of adenosine receptor oligomers were modeled via the TM4/5 interface for homodimerization, using the oligomeric structure of the β_1_-AR [PDB:4GPO] [4], or via the TM5/6 interface for heterodimerization, using the structure of the μ-OR [PDB:4DKL] [3]. The G_i_-bound A_1_R and G_s_-bound A_2A_R protomers were rotated 10° to avoid the steric clash of the N-terminal helix of G_i_ and G_s_ with the C-terminal helix (Hx8) of G_s_-unbound A_2A_R and G_i_-unbound A_1_R, respectively. This computational model, without Rluc and YFP, was placed in a rectangular box containing a lipid bilayer (814 molecules of 1-palmitoyl-2-oleoyl-sn-glycero-3-phosphocholine - POPC -) with explicit solvent (102,973 water molecules) and a 0.15 M concentration of Na^+^ and Cl^−^ (1762 ions). This initial complex was energy-minimized and subsequently subjected to a 10 ns MD equilibration, with positional restraints on protein coordinates. These restraints were released and 500 ns of MD trajectory were produced at constant pressure and temperature (see Additional file 10: Movie M1). Computer simulations were performed with the GROMACS 4.6.3 simulation package [35], using the AMBER99SB force field as implemented in GROMACS and Berger parameters for POPC lipids. This procedure has been previously validated [36].

### Availability of data and materials

The crystal structures 4EIY, 2Z73, 3SN6, 1AGR, 2RH7, 2PSD, 4GPO, and 4DKL are available from PDB (http://www.rcsb.org). All other relevant data are within the paper and its Additional files.

## Additional files

Additional file 1: Figure S1. Examples of receptor trajectories in HEK-293T cells. Images of cells expressing A_1_R-GFP (A) and of particular trajectories of A_1_R-GFP-containing (B) or A_2A_R-mCherry-containing (C) particles. (TIF 1164 kb) Additional file 2: Figure S2. Graphical description of the stoichiometry of A_1_R-GFP, A_2A_R-mCherry or both A_1_-GFP and A_2A_-mCherry. The fluorescence intensity signal distribution (gray area) detected for more than 7000 independent observations is given for HEK-293T cells expressing A_1_-GFP (A), A_2A_-mCherry (D), or both A_1_-GFP and A_2A_-mCherry (B, E). The stoichiometry analysis was performed for A_1_-GFP (A, B) and A_2A_-mCherry (D, E). Curves approximately delineating the amount of monomers, dimers, trimers, and tetramers are displayed in green for A_1_-GFP (A, B) and in red for A_2A_-mCherry (D-E). The occurrence on the cell surface of monomers, dimers, trimers, and tetramers for A_1_-GFP (C) expressed alone (black bars) or in the presence of A_2A_-mCherry (blue bars) and for A_2A_-mCherry (F) expressed alone (black bars) or in the presence of A_1_-GFP (blue bars) was calculated by stoichiometry analysis from results shown in A, B, D, and E. (TIF 455 kb) Additional file 3: Figure S3. Controls of cAMP production and BRET assays in cells expressing minigenes and in cells expressing the ghrelin GHS1a receptor instead of one of the adenosine receptors. (A,B) cAMP determination in HEK-293T cells transfected with (A) 0.3 μg of cDNA corresponding to A_1_R or (B) with 0.2 μg of cDNA corresponding to A_2A_R with (control) or without 0.5 μg of cDNA corresponding to minigenes coding for peptides blocking either Gi or Gs binding. Cells were stimulated with the A_1_R agonist N^6^-Cyclopentyladenosine (CPA) (10 nM, red bars) in the presence of 0.5 μM forskolin (Fk) or with the A_2A_R agonist 4-[2-[[6-Amino-9-(N-ethyl-β-D-ribofuranuronamidosyl)-9H-purin-2-yl]amino]ethyl]benzenepropanoic acid hydrochloride (CGS-21680) (200 nM, blue bars). Values expressed as % of the forskolin-treated cells (CPA reduces forskolin-induced cAMP levels, red bars) or of the basal (CGS 21680 *per se* enhances cAMP levels, blue bars) are given as mean ± SD (n = 4–8). One-way ANOVA followed by a Bonferroni post - hoc test showed a significant effect of CPA when compared with that of forskolin (red bars, ****p* < 0.001) or of CGS 21680 when compared to basal cAMP levels (blue bars, ^##^ *p* < 0.01, ^###^ *p* < 0.001). (C, D) BRET saturation curves were performed in HEK-293T cells transfected with (C) 0.3 μg cDNA coding for A_1_R-Rluc, increasing amounts of cDNA coding for A_1_R-YFP (0.1–1.5 μg cDNA), and 0.4 μg cDNA coding for GHS1a, or (D) with 0.2 μg of cDNA coding for A_2A_R-Rluc, increasing amounts of cDNA coding for A_2A_R-YFP (0.1–1.0 μg cDNA), and 0.5 μg cDNA coding for to GHS1a. Prior to BRET determination, cells were treated for 16 h with medium (black curves), with 10 ng/ml of pertussis toxin (green curves), or with 100 ng/ml of cholera toxin (red curves). mili BRET units (mBU) are given as the mean ± SD (n = 4–6 different experiments grouped as a function of the amount of BRET acceptor). (TIF 1418 kb) Additional file 4: Figure S4. Possible interfaces in A_2A_R homodimers in complex with G_s_. In A–E, the A_2A_R homodimer was modeled through TM4 using the H_1_-receptor structure as template (A), through TM5 using the structure of squid rhodopsin (B), through TM4/5 using the β_1_-receptor structure (C), and via TM5/6 (D) and TM1 (E) using the μ-OR structure. TM helices 1, 4, and 5 involved in receptor dimerization are highlighted in dark blue, light blue, and gray, respectively. A_2A_R protomers bound to G_s_ (in gray) are shown in light green, whereas G_s_-unbound A_2A_R protomers are shown in dark green. Rluc (blue) is attached to the N-terminal αN helix of G_s_, and YFP (yellow) is attached to the C-terminal domain of the G_s_-unbound A_2A_R protomer (light green). It is important to note that the position of YFP is highly dependent on the orientation of the long and highly flexible C-tail of A_2A_R (102 amino acids, Gln311–Ser412), which was modeled as described for the OXER [32] (see Additional file 9: Figure S9 for details). Despite these limitations, we can crudely estimate the approximate distances between the center of mass of Rluc and YFP as 4.6, 10.1, 6.5, 11.6, and 8.3 nm for panels A–E, respectively. Thus, among all these possible dimeric interfaces, only the molecular models depicted in panels A (TM4 interface) and C (TM4/5 interface) would favor the observed high-energy transfer between G_s_-Rluc and A_2A_R-YFP (Fig. 4a in main paper). However, there is a steric clash between the N-terminal helix of Gs and the dark-green protomer in the TM4 interface. Accordingly, we have modeled A_2A_R homodimerization via the TM4/5 interface. Unfortunately, similar experiments with cells transfected with G_i_-Rluc and A_1_R-YFP could not be accomplished because of a lack of receptor expression (not shown); it is likely that the shorter C-tail of A_1_R (16 amino acids, Pro311–Asp326) could not accommodate YFP in the presence of G_i_ in the right three-dimensional structure. The A_1_R homodimer was built using the same TM4/5 interface as for A_2A_R. (TIF 3135 kb) Additional file 5: Figure S5. BRET assays in cells expressing fusion proteins containing hemi-Rluc8 and hemi-Venus moieties fused to adenosine receptors or containing the ghrelin GHS1a receptor instead of one of the adenosine receptors. (A) Saturation BRET curve in HEK-293T co-transfected with 1.5 μg of the two cDNAs corresponding to A_1_R-cRLuc8 and A_2A_R-nRLuc8 and with increasing amounts of cDNAs corresponding to A_1_R-nVenus and A_2A_R-cVenus (equal amounts of the two cDNAs). BRET_max_ was 35 ± 2 mBU and BRET_50_ was 16 ± 3 mBU. BRET in cells expressing cRluc8 instead of A_1_R-cRluc8 gave a linear, non-saturable signal. (B) Comparison of BRET responses using complementary and non-complementary pairs, or replacing one adenosine receptor with the ghrelin GHS1a (gn) receptor. Data are mean ± SD of three different experiments grouped as a function of the amount of BRET acceptor. ****p* < 0.001 with respect to BRET in cells expressing adenosine receptors and hemi-Rluc8 and hemi-Venus proteins. (TIF 398 kb) Additional file 6: Figure S6. Details of the relative position of Rluc and YFP in a receptor heterotetramer interacting with two G proteins. Computational-based model of G_s_ and G_i_ bound to the adenosine A_1_R-A_2A_R heterotetramer. Rluc and YFP fused to the N-terminal domain of the G_α_-subunits point toward different positions in space (A), whereas Rluc and YFP fused to G_**γ**_-subunits are close (B). The color code of the proteins is depicted in the adjacent schematic representations (TM4 and TM5 of GPCR protomers are in light blue and gray, respectively). (TIF 6445 kb) Additional file 7: Figure S7. Molecular dynamics (MD) simulation of the adenosine A_1_R-A_2A_R heterotetramer in complex with G_i_ and G_s_. (A) Root-mean-square deviations (rmsd) on protein α-carbons of the whole system (black solid line), of the two A_1_Rs (orange and red solid lines), of the two A_2A_Rs (light and dark green solid lines), of G_i_ (gray solid line), and of G_s_ (gray dotted line) throughout the MD simulation. This color scheme matches with the color of the different proteins depicted in the two adjacent schematic representations. (B) Intermolecular distances between the N-terminal helices of the γ-subunit of Gi and Gs (magenta line), the N-terminal helices of the α-subunit of G_i_ and G_s_ (gray line), the N-terminal helix of the α-subunit of G_i_ and the C-terminal helix (Hx8) of inactive A_1_R (orange line), the N-terminal helix of the α-subunit of G_s_ and the C-terminal Hx8 of inactive A_2A_R (green line), the C-terminal Hx8 of A_1_R and A_2A_R (blue lines). These computed intermolecular distances are depicted as double arrows in the two adjacent schematic representations. (TIF 6973 kb) Additional file 8: Figure S8. Evolution of TM4/5 and TM5/6 interfaces as devised from MD simulations of the adenosine A_1_R-A_2A_R heterotetramer in complex with G_i_ and G_s_. (A) Representative snapshots (20 structures collected every 25 ns) of the TM domains of A_1_R bound to G_i_ (red), G_i_-unbound A_1_R (orange), A_2A_R bound to G_s_ (dark green), and G_s_-unbound A_2A_R (light green). TM helices 4 and 5 are highlighted in light blue and gray, respectively. Initial (at 0 ns, transparent cylinders) and final (at 500 ns, solid cylinders) snapshots of TM interfaces are shown for homodimerization (TM4/5, within rectangles) and heterodimerization (TM5/6, within a circle) bundles. TM helices 4 (light blue), 5 (gray), and 6 (orange and green) are highlighted. (B) Root-mean-square deviations (rmsd) on protein α-carbons of the four-helix bundles forming the TM5/6 interface (orange solid line), TM4/5 interface of A_1_R (blue dotted line), and TM4/5 interface of A_2A_R (blue solid line) throughout the MD simulation. (C) Contact maps of the TM4/5 interface (rectangles in panel A) in the A_1_R or A_2A_R homodimer (left and right panels) and of the TM5/6 interface (circle in panel A) in the A_1_R-A_2A_R heterodimer (middle panel). Darker dots show more frequent contacts. (D) Detailed view of the extensive network of hydrophobic interactions (mainly of aromatic side chains) within the TM4/5 (left and right panels) and TM5/6 (middle panel) interfaces. The amino acids are numbered following the generalized numbering scheme of Ballesteros and Weinstein [37, 38]. This allows easy comparison among residues in the 7TM segments of different receptors. (TIF 4004 kb) Additional file 9: Figure S9. Positioning YFP in the C-tail of A_2A_R. The complex between the A_2A_R protomer (in light green) and G_s_ (α-subunit in dark grey and yellow, β-subunit in light gray, and γ-subunit in purple) was constructed from the crystal structure of β_2_ in complex with G_s_ [33]. Although the exact conformation of the A_2A_R C-tail (102 amino acids, Gln311–Ser412) cannot unambiguously be determined, its orientation was modeled as in the C-tail of squid rhodopsin [39], which contains the conserved amphipathic helix 8 that runs parallel to the membrane and an additional cytoplasmic helix 9. Thus, the C-tail of A_2A_R expands (see solid light green line) and points intracellularly toward the N-termini of the γ-subunit as suggested for OXER [32]. The laboratory of Kostenis has shown that the C-terminal of OXER, labeled with Rluc (OXER-Rluc), gets close to the N-terminal of the γ-subunit, labeled with GFP (γ-GFP) [32]. Analogously, we propose that YFP attached to the C-tail of A_2A_R is positioned near the N-termini of the γ-subunit (in purple). (TIF 2395 kb) Additional file 10: Movie M1. Assembly of adenosine A_1_ and A_2A_ receptors in complex with two G proteins and MD simulation of the system. The assembly of G_s_ and G_i_ bound to the adenosine A_1_R-A_2A_R heterotetramer was subjected to 500 ns of MD simulation in a rectangular box containing the system, the lipid bilayer, explicit solvent, and ions. A_1_R protomers are in orange and red, A_2A_R protomers in light and dark green, G_α_ in white, G_β_ in gray, and G_γ_ in purple. For easier visualization of protomer-protomer interfaces, TMs 4 and 5 are highlighted in blue and white, respectively. (MPEG 87870 kb) Additional file 11: Supplementary methods. (DOCX 72 kb)
