# Supplementary figures and images for: Quaternary structure of a G-protein-coupled receptor heterotetramer in complex with Gi and Gs
Source: BMC Biol. 2016 Apr 5;14:26. doi: 10.1186/s12915-016-0247-4 (PMC4822319; doi:10.1186/s12915-016-0247-4)

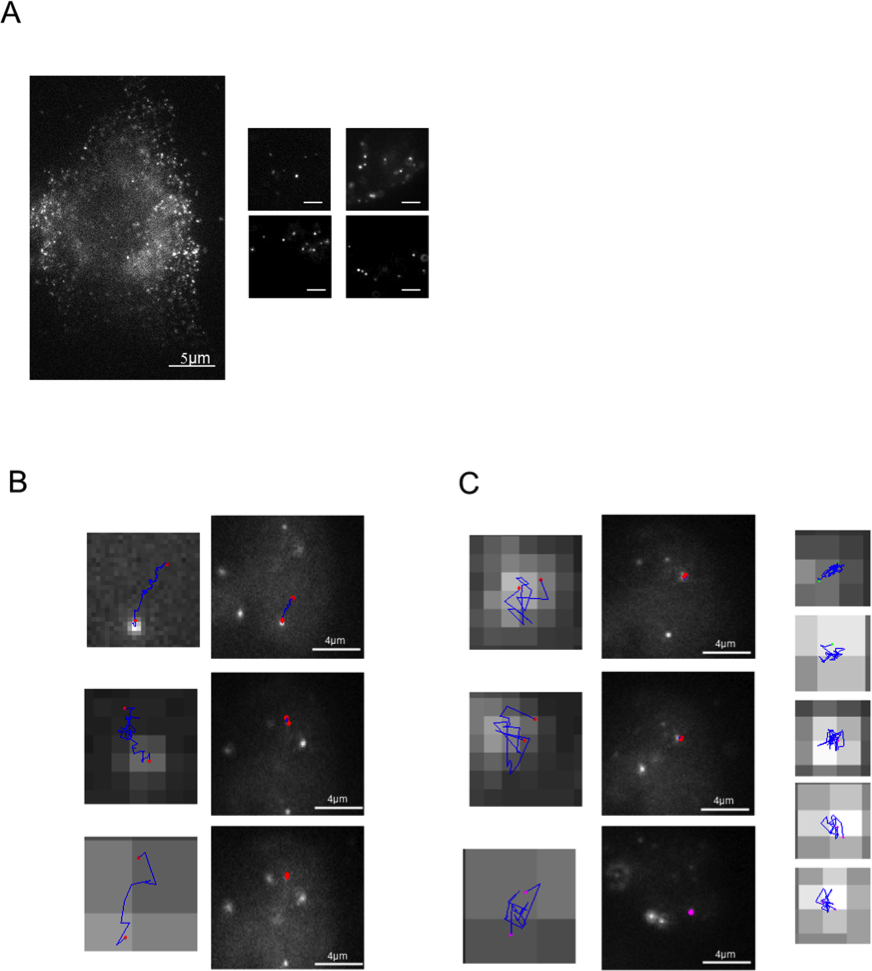

Supplement: Additional file 1: Figure S1. — Examples of receptor trajectories in HEK-293T cells. Images of cells expressing A1R-GFP (A) and of particular trajectories of A1R-GFP-containing (B) or A2AR-mCherry-containing (C) particles. (TIF 1164 kb) [file 12915_2016_247_MOESM1_ESM.tif]

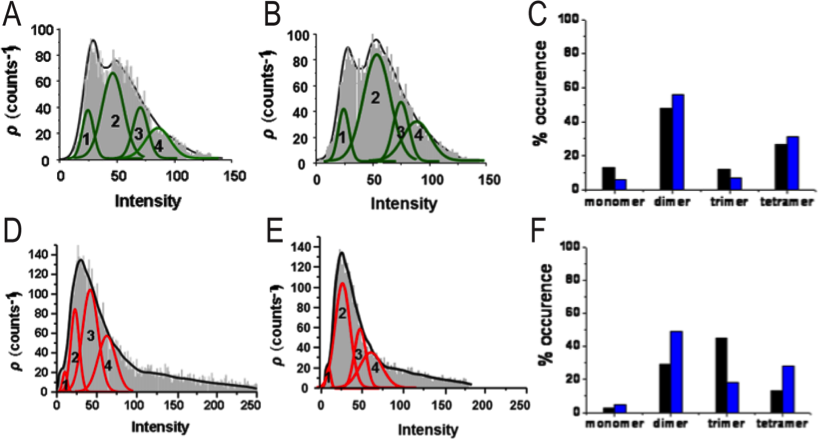

Supplement: Additional file 2: Figure S2. — Graphical description of the stoichiometry of A1R-GFP, A2AR-mCherry or both A1-GFP and A2A-mCherry. The fluorescence intensity signal distribution (gray area) detected for more than 7000 independent observations is given for HEK-293T cells expressing A1-GFP (A), A2A-mCherry (D), or both A1-GFP and A2A-mCherry (B, E). The stoichiometry analysis was performed for A1-GFP (A, B) and A2A-mCherry (D, E). Curves approximately delineating the amount of monomers, dimers, trimers, and tetramers are displayed in green for A1-GFP (A, B) and in red for A2A-mCherry (D-E). The occurrence on the cell surface of monomers, dimers, trimers, and tetramers for A1-GFP (C) expressed alone (black bars) or in the presence of A2A-mCherry (blue bars) and for A2A-mCherry (F) expressed alone (black bars) or in the presence of A1-GFP (blue bars) was calculated by stoichiometry analysis from results shown in A, B, D, and E. (TIF 455 kb) [file 12915_2016_247_MOESM2_ESM.tif]

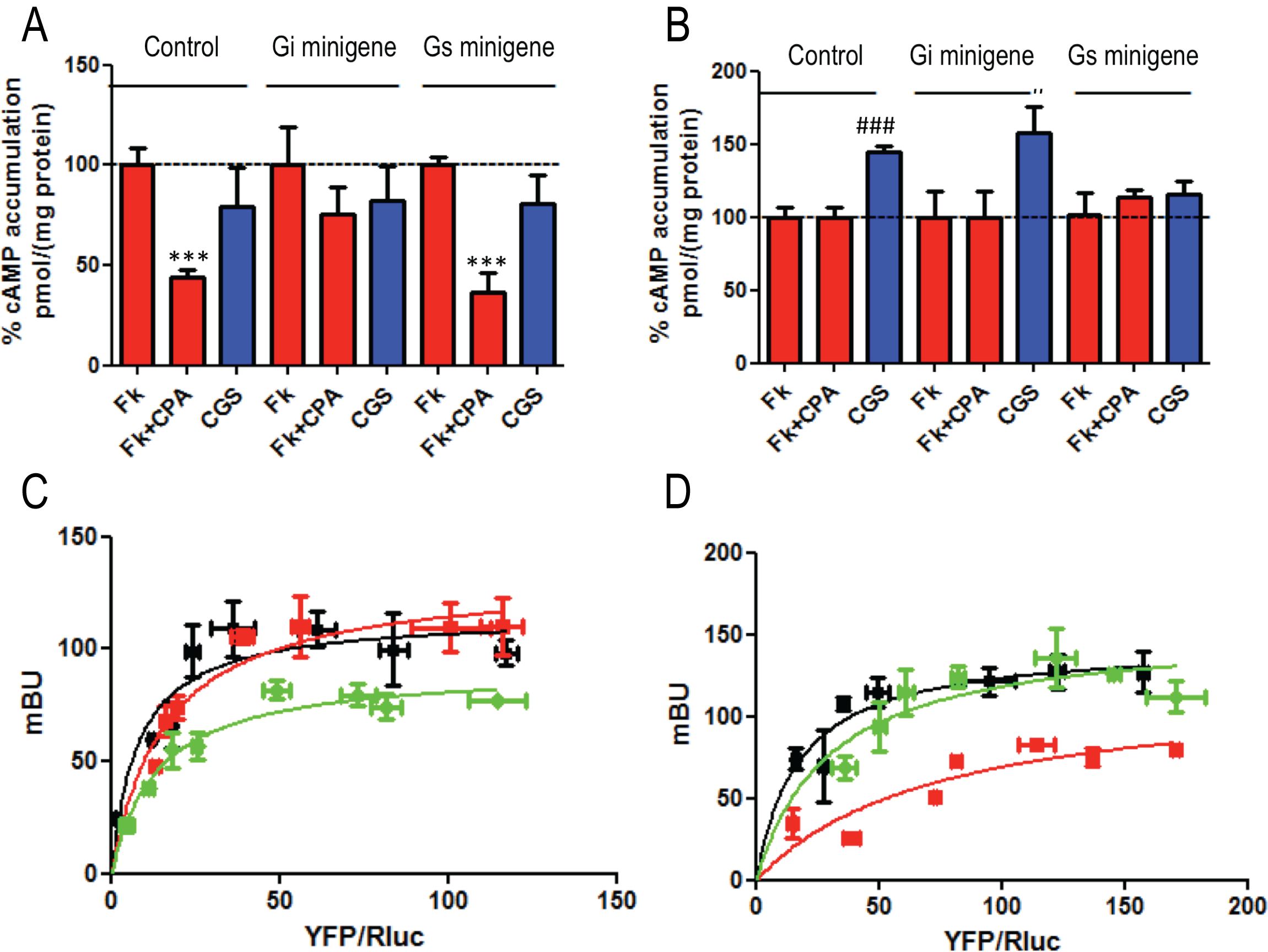

Supplement: Additional file 3: Figure S3. — Controls of cAMP production and BRET assays in cells expressing minigenes and in cells expressing the ghrelin GHS1a receptor instead of one of the adenosine receptors. (A,B) cAMP determination in HEK-293T cells transfected with (A) 0.3 μg of cDNA corresponding to A1R or (B) with 0.2 μg of cDNA corresponding to A2AR with (control) or without 0.5 μg of cDNA corresponding to minigenes coding for peptides blocking either Gi or Gs binding. Cells were stimulated with the A1R agonist N6-Cyclopentyladenosine (CPA) (10 nM, red bars) in the presence of 0.5 μM forskolin (Fk) or with the A2AR agonist 4-[2-[[6-Amino-9-(N-ethyl-β-D-ribofuranuronamidosyl)-9H-purin-2-yl]amino]ethyl]benzenepropanoic acid hydrochloride (CGS-21680) (200 nM, blue bars). Values expressed as % of the forskolin-treated cells (CPA reduces forskolin-induced cAMP levels, red bars) or of the basal (CGS 21680 per se enhances cAMP levels, blue bars) are given as mean ± SD (n = 4–8). One-way ANOVA followed by a Bonferroni post - hoc test showed a significant effect of CPA when compared with that of forskolin (red bars, ***p < 0.001) or of CGS 21680 when compared to basal cAMP levels (blue bars, ## p < 0.01, ### p < 0.001). (C, D) BRET saturation curves were performed in HEK-293T cells transfected with (C) 0.3 μg cDNA coding for A1R-Rluc, increasing amounts of cDNA coding for A1R-YFP (0.1–1.5 μg cDNA), and 0.4 μg cDNA coding for GHS1a, or (D) with 0.2 μg of cDNA coding for A2AR-Rluc, increasing amounts of cDNA coding for A2AR-YFP (0.1–1.0 μg cDNA), and 0.5 μg cDNA coding for to GHS1a. Prior to BRET determination, cells were treated for 16 h with medium (black curves), with 10 ng/ml of pertussis toxin (green curves), or with 100 ng/ml of cholera toxin (red curves). mili BRET units (mBU) are given as the mean ± SD (n = 4–6 different experiments grouped as a function of the amount of BRET acceptor). (TIF 1418 kb) [file 12915_2016_247_MOESM3_ESM.tif]

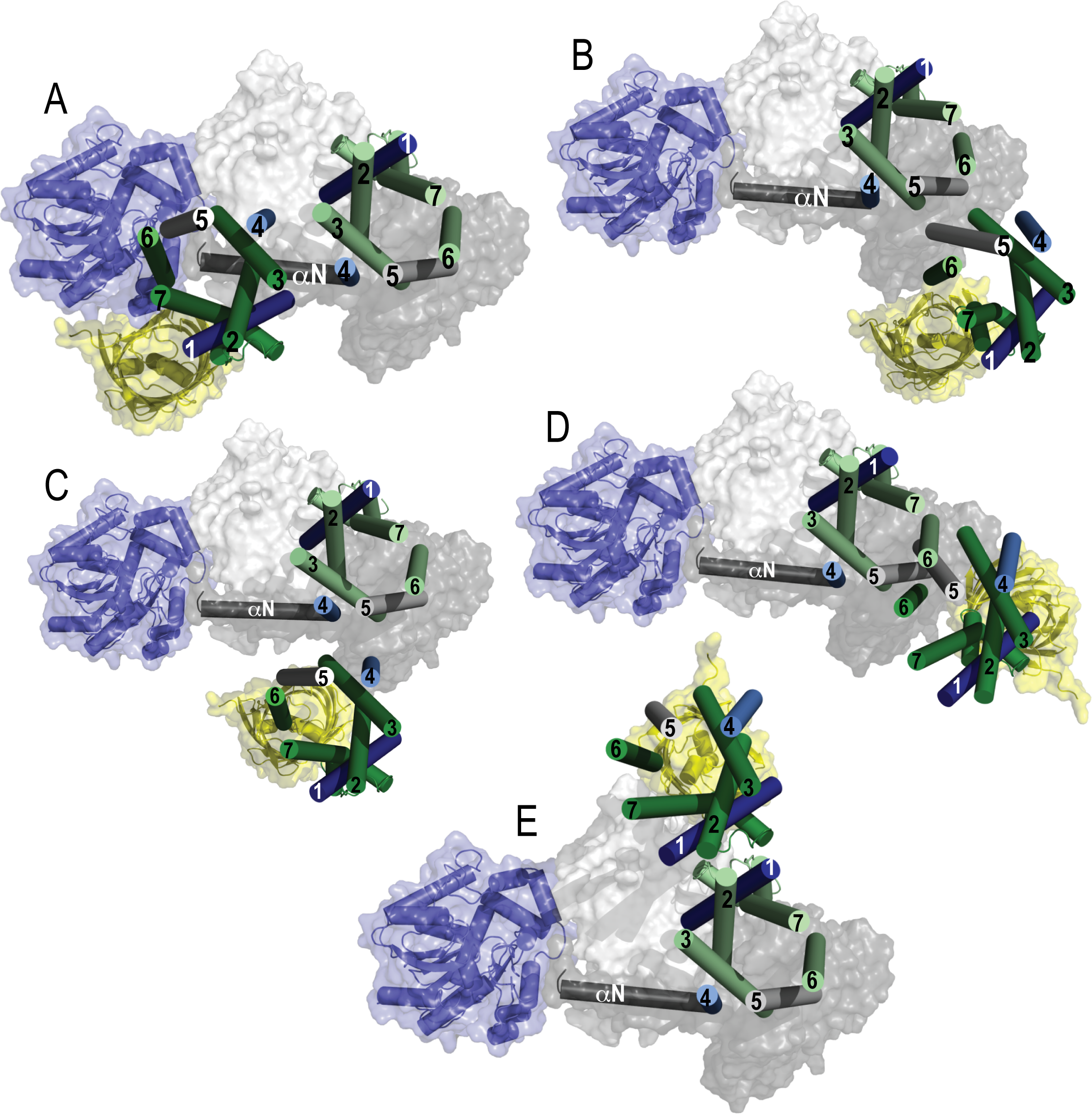

Supplement: Additional file 4: Figure S4. — Possible interfaces in A2AR homodimers in complex with Gs. In A–E, the A2AR homodimer was modeled through TM4 using the H1-receptor structure as template (A), through TM5 using the structure of squid rhodopsin (B), through TM4/5 using the β1-receptor structure (C), and via TM5/6 (D) and TM1 (E) using the μ-OR structure. TM helices 1, 4, and 5 involved in receptor dimerization are highlighted in dark blue, light blue, and gray, respectively. A2AR protomers bound to Gs (in gray) are shown in light green, whereas Gs-unbound A2AR protomers are shown in dark green. Rluc (blue) is attached to the N-terminal αN helix of Gs, and YFP (yellow) is attached to the C-terminal domain of the Gs-unbound A2AR protomer (light green). It is important to note that the position of YFP is highly dependent on the orientation of the long and highly flexible C-tail of A2AR (102 amino acids, Gln311–Ser412), which was modeled as described for the OXER [32] (see Additional file 9: Figure S9 for details). Despite these limitations, we can crudely estimate the approximate distances between the center of mass of Rluc and YFP as 4.6, 10.1, 6.5, 11.6, and 8.3 nm for panels A–E, respectively. Thus, among all these possible dimeric interfaces, only the molecular models depicted in panels A (TM4 interface) and C (TM4/5 interface) would favor the observed high-energy transfer between Gs-Rluc and A2AR-YFP (Fig. 4a in main paper). However, there is a steric clash between the N-terminal helix of Gs and the dark-green protomer in the TM4 interface. Accordingly, we have modeled A2AR homodimerization via the TM4/5 interface. Unfortunately, similar experiments with cells transfected with Gi-Rluc and A1R-YFP could not be accomplished because of a lack of receptor expression (not shown); it is likely that the shorter C-tail of A1R (16 amino acids, Pro311–Asp326) could not accommodate YFP in the presence of Gi in the right three-dimensional structure. The A1R homodimer was built using the same TM4/5 interface as [file 12915_2016_247_MOESM4_ESM.tif]

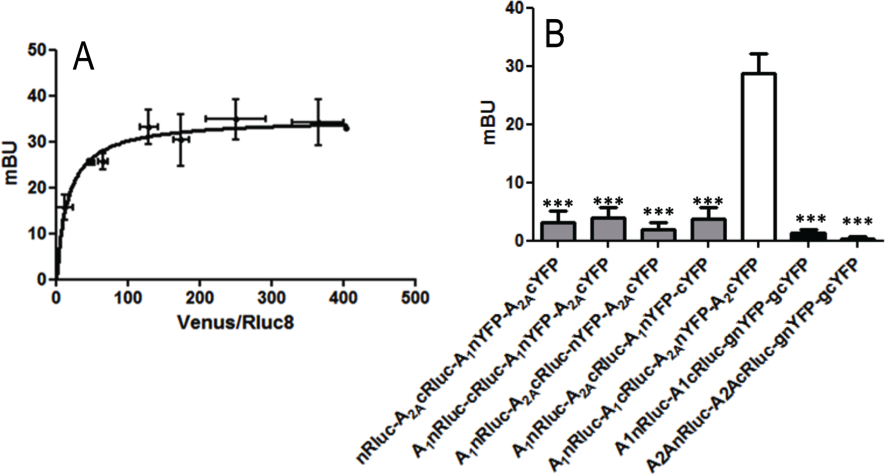

Supplement: Additional file 5: Figure S5. — BRET assays in cells expressing fusion proteins containing hemi-Rluc8 and hemi-Venus moieties fused to adenosine receptors or containing the ghrelin GHS1a receptor instead of one of the adenosine receptors. (A) Saturation BRET curve in HEK-293T co-transfected with 1.5 μg of the two cDNAs corresponding to A1R-cRLuc8 and A2AR-nRLuc8 and with increasing amounts of cDNAs corresponding to A1R-nVenus and A2AR-cVenus (equal amounts of the two cDNAs). BRETmax was 35 ± 2 mBU and BRET50 was 16 ± 3 mBU. BRET in cells expressing cRluc8 instead of A1R-cRluc8 gave a linear, non-saturable signal. (B) Comparison of BRET responses using complementary and non-complementary pairs, or replacing one adenosine receptor with the ghrelin GHS1a (gn) receptor. Data are mean ± SD of three different experiments grouped as a function of the amount of BRET acceptor. ***p < 0.001 with respect to BRET in cells expressing adenosine receptors and hemi-Rluc8 and hemi-Venus proteins. (TIF 398 kb) [file 12915_2016_247_MOESM5_ESM.tif]

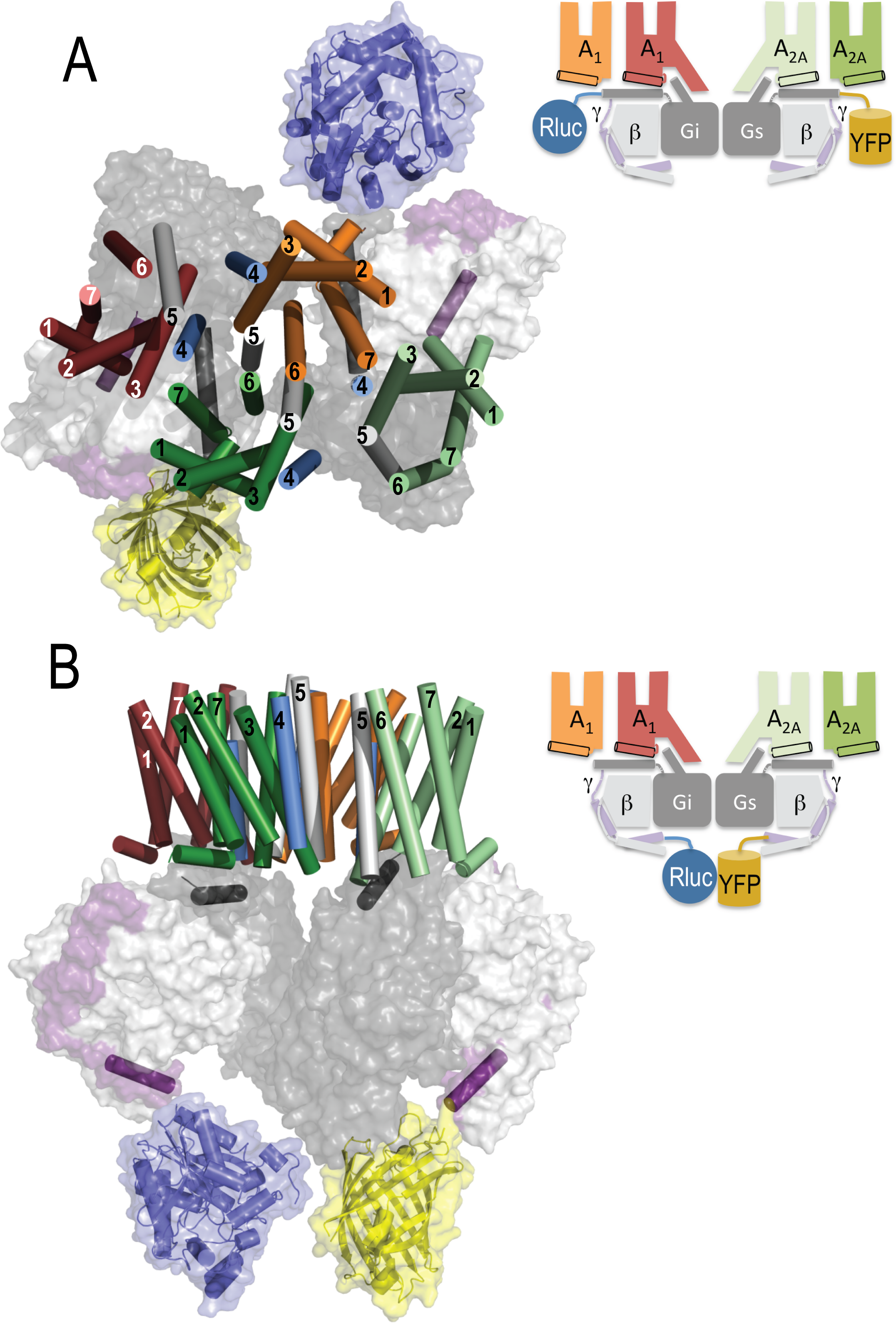

Supplement: Additional file 6: Figure S6. — Details of the relative position of Rluc and YFP in a receptor heterotetramer interacting with two G proteins. Computational-based model of Gs and Gi bound to the adenosine A1R-A2AR heterotetramer. Rluc and YFP fused to the N-terminal domain of the Gα-subunits point toward different positions in space (A), whereas Rluc and YFP fused to Gγ-subunits are close (B). The color code of the proteins is depicted in the adjacent schematic representations (TM4 and TM5 of GPCR protomers are in light blue and gray, respectively). (TIF 6445 kb) [file 12915_2016_247_MOESM6_ESM.tif]

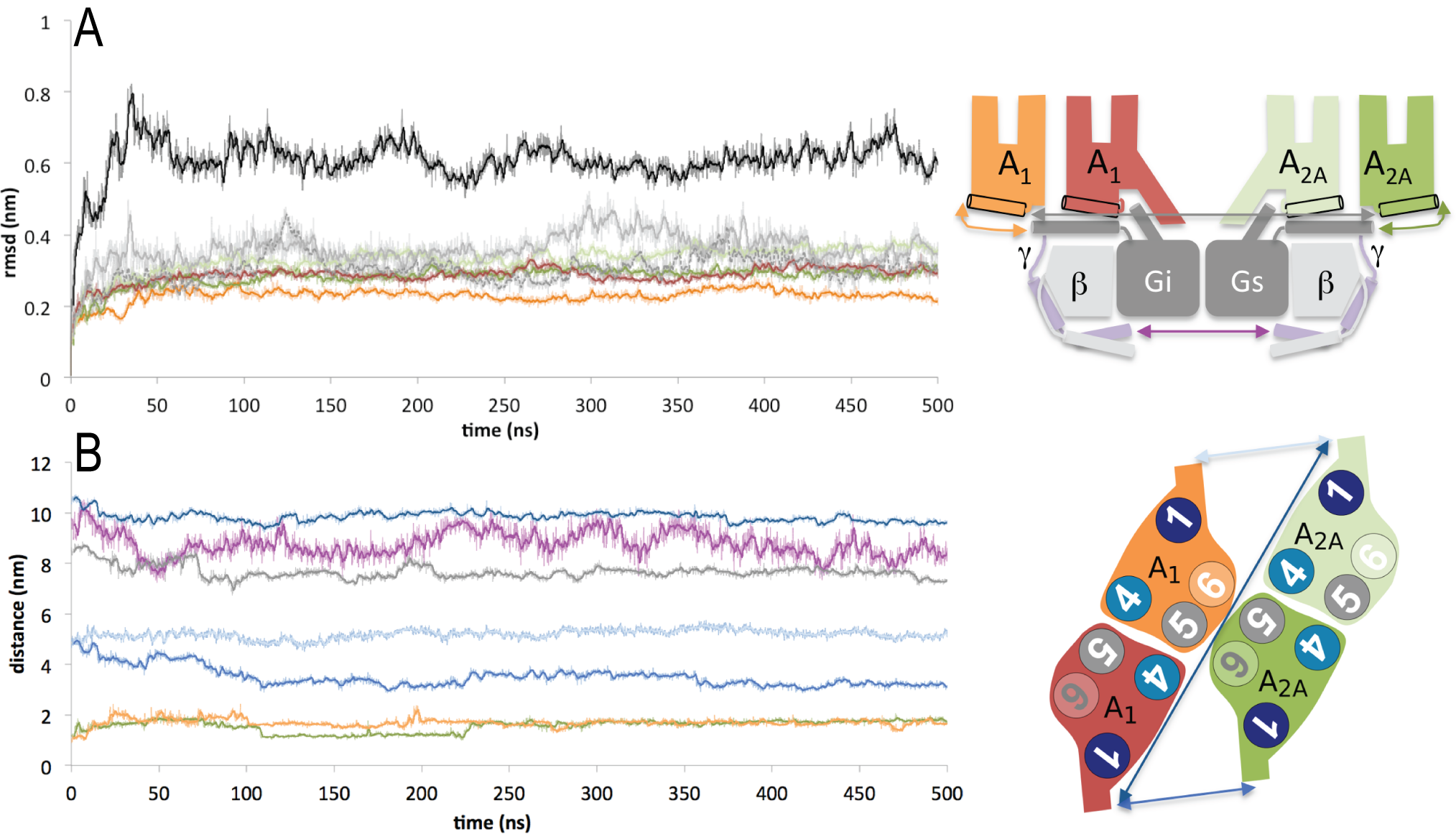

Supplement: Additional file 7: Figure S7. — Molecular dynamics (MD) simulation of the adenosine A1R-A2AR heterotetramer in complex with Gi and Gs. (A) Root-mean-square deviations (rmsd) on protein α-carbons of the whole system (black solid line), of the two A1Rs (orange and red solid lines), of the two A2ARs (light and dark green solid lines), of Gi (gray solid line), and of Gs (gray dotted line) throughout the MD simulation. This color scheme matches with the color of the different proteins depicted in the two adjacent schematic representations. (B) Intermolecular distances between the N-terminal helices of the γ-subunit of Gi and Gs (magenta line), the N-terminal helices of the α-subunit of Gi and Gs (gray line), the N-terminal helix of the α-subunit of Gi and the C-terminal helix (Hx8) of inactive A1R (orange line), the N-terminal helix of the α-subunit of Gs and the C-terminal Hx8 of inactive A2AR (green line), the C-terminal Hx8 of A1R and A2AR (blue lines). These computed intermolecular distances are depicted as double arrows in the two adjacent schematic representations. (TIF 6973 kb) [file 12915_2016_247_MOESM7_ESM.tif]

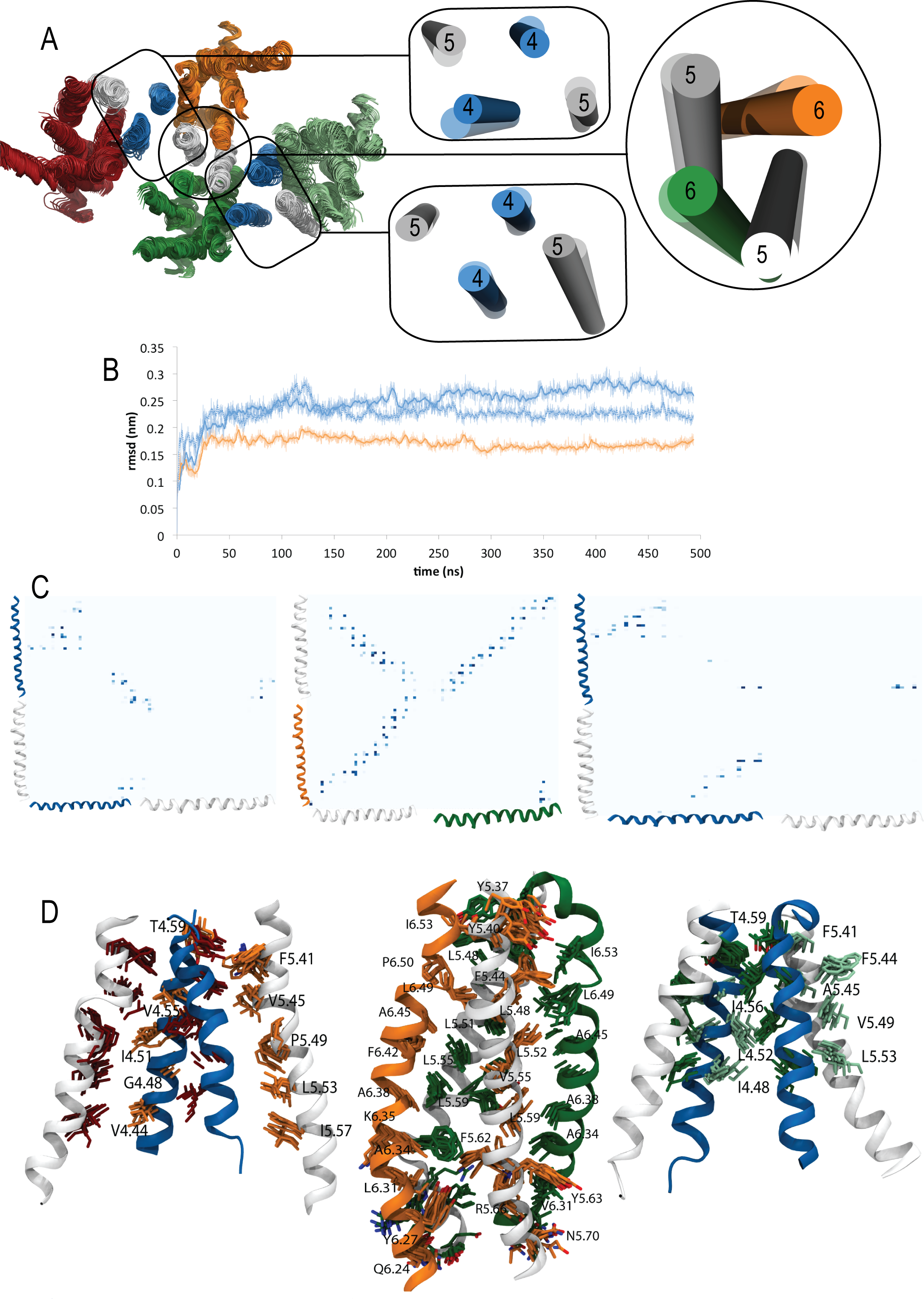

Supplement: Additional file 8: Figure S8. — Evolution of TM4/5 and TM5/6 interfaces as devised from MD simulations of the adenosine A1R-A2AR heterotetramer in complex with Gi and Gs. (A) Representative snapshots (20 structures collected every 25 ns) of the TM domains of A1R bound to Gi (red), Gi-unbound A1R (orange), A2AR bound to Gs (dark green), and Gs-unbound A2AR (light green). TM helices 4 and 5 are highlighted in light blue and gray, respectively. Initial (at 0 ns, transparent cylinders) and final (at 500 ns, solid cylinders) snapshots of TM interfaces are shown for homodimerization (TM4/5, within rectangles) and heterodimerization (TM5/6, within a circle) bundles. TM helices 4 (light blue), 5 (gray), and 6 (orange and green) are highlighted. (B) Root-mean-square deviations (rmsd) on protein α-carbons of the four-helix bundles forming the TM5/6 interface (orange solid line), TM4/5 interface of A1R (blue dotted line), and TM4/5 interface of A2AR (blue solid line) throughout the MD simulation. (C) Contact maps of the TM4/5 interface (rectangles in panel A) in the A1R or A2AR homodimer (left and right panels) and of the TM5/6 interface (circle in panel A) in the A1R-A2AR heterodimer (middle panel). Darker dots show more frequent contacts. (D) Detailed view of the extensive network of hydrophobic interactions (mainly of aromatic side chains) within the TM4/5 (left and right panels) and TM5/6 (middle panel) interfaces. The amino acids are numbered following the generalized numbering scheme of Ballesteros and Weinstein [37, 38]. This allows easy comparison among residues in the 7TM segments of different receptors. (TIF 4004 kb) [file 12915_2016_247_MOESM8_ESM.tif]

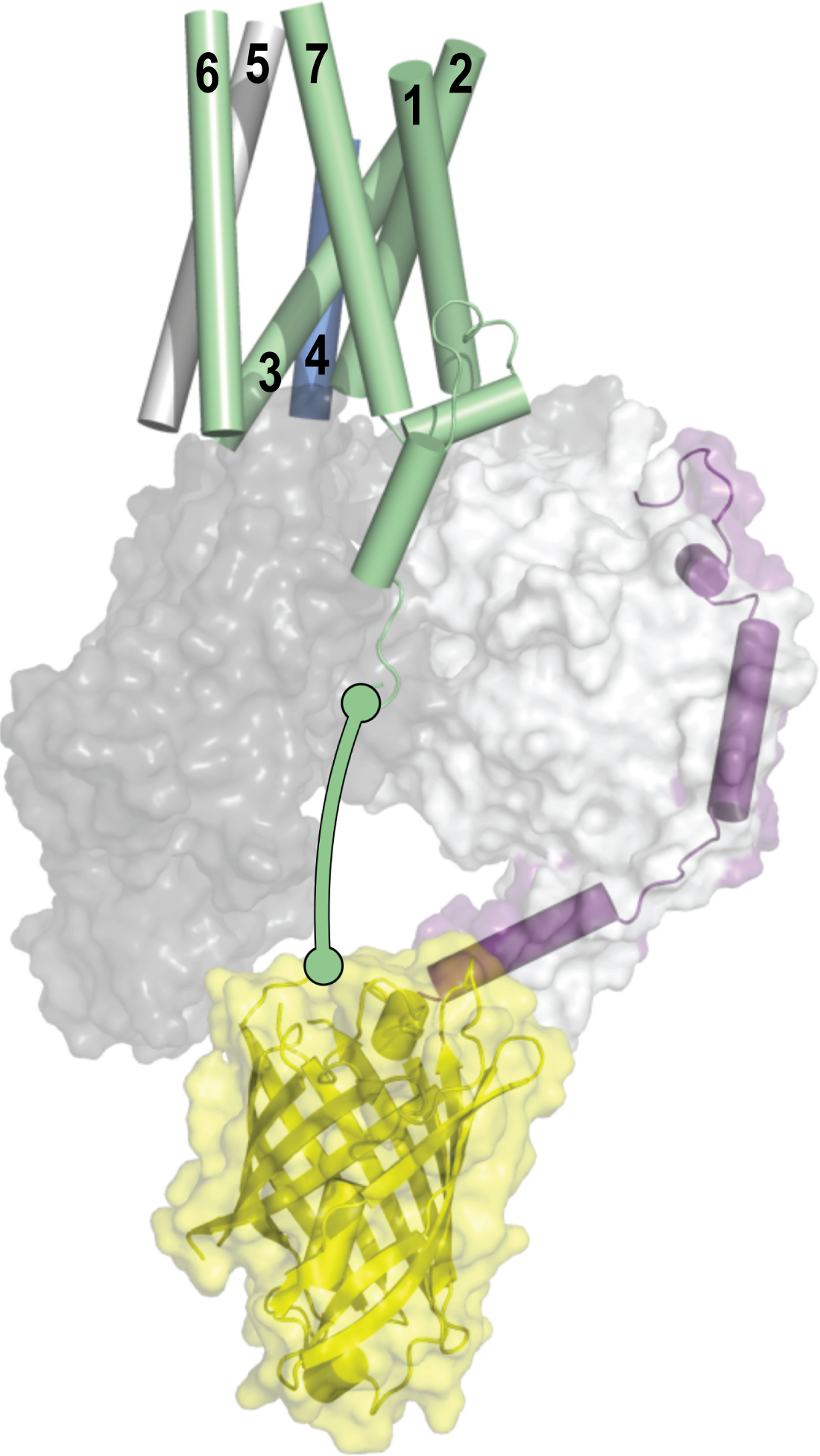

Supplement: Additional file 9: Figure S9. — Positioning YFP in the C-tail of A2AR. The complex between the A2AR protomer (in light green) and Gs (α-subunit in dark grey and yellow, β-subunit in light gray, and γ-subunit in purple) was constructed from the crystal structure of β2 in complex with Gs [33]. Although the exact conformation of the A2AR C-tail (102 amino acids, Gln311–Ser412) cannot unambiguously be determined, its orientation was modeled as in the C-tail of squid rhodopsin [39], which contains the conserved amphipathic helix 8 that runs parallel to the membrane and an additional cytoplasmic helix 9. Thus, the C-tail of A2AR expands (see solid light green line) and points intracellularly toward the N-termini of the γ-subunit as suggested for OXER [32]. The laboratory of Kostenis has shown that the C-terminal of OXER, labeled with Rluc (OXER-Rluc), gets close to the N-terminal of the γ-subunit, labeled with GFP (γ-GFP) [32]. Analogously, we propose that YFP attached to the C-tail of A2AR is positioned near the N-termini of the γ-subunit (in purple). (TIF 2395 kb) [file 12915_2016_247_MOESM9_ESM.tif]
