# Supplementary material for: Quaternary structure of a G-protein-coupled receptor heterotetramer in complex with Gi and Gs
Source: BMC Biol. 2016 Apr 5;14:26. doi: 10.1186/s12915-016-0247-4 (PMC4822319; doi:10.1186/s12915-016-0247-4)
Supplement: Additional file 11: — Supplementary methods. (DOCX 72 kb) [file 12915_2016_247_MOESM11_ESM.docx]

**SUPPLEMENTARY METHODS**

Quaternary structure of a G-protein-coupled receptor heterotetramer coupled to Gi and Gs

Gemma Navarro^1,2,3^, Arnau Cordomí^4^, Monika Zelman-Femiak^5,6^, Marc Brugarolas^1,2,3^, Estefania Moreno^1,2,3^, David Aguinaga^1,2,3^, Laura Perez-Benito^4^, Antoni Cortés^1,2,3^, Vicent Casadó^1,2,3^, Josefa Mallol^1,2,3^, Enric I. Canela^1,2,3^, Carme Lluís^1,2,3^, Leonardo Pardo^4^, Ana J. García-Saez^5,6^, Peter J. McCormick^1,2,3^ and Rafael Franco^1,2,3^

1 Centro de Investigación Biomédica en Red sobre Enfermedades Neurodegenerativas.

2 Institute of Biomedicine of the University of Barcelona (IBUB).

3 Department of Biochemistry and Molecular Biology, Faculty of Biology, University of Barcelona, Barcelona, 08028 Spain;

4 Laboratori de Medicina Computacional, Unitat de Bioestadística, Facultat de Medicina, Universitat Autònoma de Barcelona, 08193 Bellaterra, Spain;

5 Max Planck Institute for Intelligent Systems, Heisenbergstr. 3, 70569 Stuttgart;

6 German Cancer Research Center, Bioquant, Im Neuenheimer Feld 267, 69120 Heidelberg;

7 Interfaculty Institute of Biochemistry, Hoppe-Seyler-Str. 4, 72076 Tübingen;

8 School of Pharmacy, University of East Anglia, Norwich NR4 7TJ.

*These authors contributed equally to this work

^&^These authors contributed equally to this work

Corresponding authors:

Peter J. McCormick: p.mccormick@uea.ac.uk, Rafael Franco: rfranco@ub.edu

KEYWORDS. GPCR • heterotetramer • single-particle tracking • BRET • molecular dynamics

**Single particle data analysis**

Data processing was performed using Matlab (MathWorks, Natick, MA, USA). By correlation analysis between consecutive images the two dimensional trajectories of individual molecules in the plane of focus were reconstructed by determining the probability and setting a high-confidence threshold that each step in a trajectory was from the same particle. Multiple data sets were produced for every receptor type and for the existing complexes of the receptors separately. In brief, trajectories were then analyzed as described previously [[1](#_ENREF_1)]. For the analysis of the (*r_i_* ^2^, *t_lag_*) plots, a positional accuracy of 14 ± 3 nm was considered in our measurements [[2](#_ENREF_2)].

The lateral diffusion of Brownian particles in a medium characterized by a diffusion constant *D* is described by the cumulative probability distribution function for the square displacements, *r* [[2](#_ENREF_2), [3](#_ENREF_3)]:


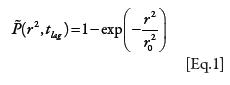


*P*(*r*^2^, *t_lag_*) is the probability that the Brownian particle starting at the origin will be found within a circle of radius *r* at time *t_lag_*. Provided that the system under study segregates into two components, characterized by mean-square displacements *r_1_* ^2^ and *r_2_* ^2^, and relative fractions α and (1 – α), respectively, equation 1 becomes [[1](#_ENREF_1), [3](#_ENREF_3)]:


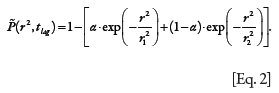


The cumulative probability distributions *P*(*r_i_* ^2^, *t_lag_*) were constructed for each time lag from the single-molecule trajectories by counting the number of square displacements with values <*r*^2^, and subsequent normalization by the total number of data points [[3](#_ENREF_3)]. Probability distributions with *n* >1,000 data points were least-square fitted to equation 2, resulting in a parameter set {*r_1_* ^2^(*t_lag_*), *r_2_* ^2^(*t_lag_*), α}, for each time lag, *t_lag_*. This approach of fitting leads to a robust estimation of the mean-square displacements *r_i_* ^2^ even when the mobility is not purely random [[1](#_ENREF_1)]. An example of the data is provided in Graph 1.


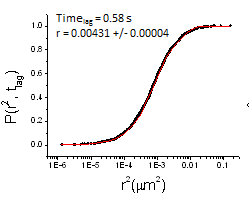


**Graph 1.** Example of the mobility probability distribution. Example from data using cells expressing A_1_R- eGFP at *t_lag_* = 0.58 s

For mobility analysis, the diffusional behavior of the respective populations of molecules was revealed by plotting the mean square displacement (*r_i_* ^2^) versus *t_lag_*. The (*r_i_* ^2^, *t_lag_*) data sets were fitted by a free diffusion model,


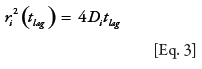


where *r_i_* ^2^ is proportional to time *t_lag_*. When diffusion is hindered by obstruction or trapping in such a way that the mean square displacement is proportional to some power of time <1 (*r_i_* ^2^ ~ *t*^α^ , α < 1) (anomalous subdiffusion), the diffusion constant becomes [[1](#_ENREF_1)]:


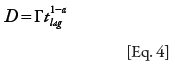


If α = 1, then *r_i_* ^2^ ~ 1, *D* = Γ is constant and diffusion is normal. The confined diffusion model assumes that diffusion is free within a square of side length *L*, surrounded by an impermeable, reflecting barrier. Then the mean-square displacement depends on *L* and the initial diffusion coefficient *D*_0_, and varies with *t_lag_* as [[1](#_ENREF_1), [4](#_ENREF_4)]:


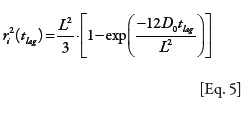


Fluorescence distribution may be used to determine local stoichiometry [[5](#_ENREF_5), [6](#_ENREF_6)]. The only difference between fluorescence of small fluorophore clusters and single fluorophores is the higher intensity. Fluorophore photobleaching or blinking has a significant impact on an average intensity of a fluorophore cluster, reducing it and this way making a direct fluorescence intensity count more complicated. The probability density function of the fluorescence intensity displays a discrete structure and may be fitted with multiple Gaussians models to calculate the molecular stoichiometry [[5](#_ENREF_5), [6](#_ENREF_6)].

REFERENCES FOR SUPPLEMENTARY METHODS

1. Lommerse PH, Blab GA, Cognet L, Harms GS, Snaar-Jagalska BE, Spaink HP, Schmidt T: Single-molecule imaging of the H-ras membrane-anchor reveals domains in the cytoplasmic leaflet of the cell membrane. *Biophys J* 2004, 86:609-616.

2. Thompson RE, Larson DR, Webb WW: Precise nanometer localization analysis for individual fluorescent probes. *Biophys J* 2002, 82:2775-2783.

3. Schutz GJ, Schindler H, Schmidt T: Single-molecule microscopy on model membranes reveals anomalous diffusion. *Biophys J* 1997, 73:1073-1080.

4. Kusumi A, Nakada C, Ritchie K, Murase K, Suzuki K, Murakoshi H, Kasai RS, Kondo J, Fujiwara T: Paradigm shift of the plasma membrane concept from the two-dimensional continuum fluid to the partitioned fluid: high-speed single-molecule tracking of membrane molecules. *Annu Rev BiophysBiomol Struct* 2005, 34:351-378.

5. Schmidt T, Schütz GJ, Gruber HJ, Schindler H: Local Stoichiometries Determined by Counting Individual Molecules. *Anal Chem* 1996, 68:4397-4401.

6. Harms GS, Cognet L, Lommerse PH, Blab GA, Kahr H, Gamsjager R, Spaink HP, Soldatov NM, Romanin C, Schmidt T: Single-molecule imaging of l-type Ca(2+) channels in live cells. *Biophys J* 2001, 81:2639-2646.
